# Transcriptome Analysis Reveals Regulation of Gene Expression for Lipid Catabolism in Young Broilers by Butyrate Glycerides

**DOI:** 10.1371/journal.pone.0160751

**Published:** 2016-08-10

**Authors:** Fugui Yin, Hai Yu, Dion Lepp, Xuejiang Shi, Xiaojian Yang, Jielun Hu, Steve Leeson, Chengbo Yang, Shaoping Nie, Yongqing Hou, Joshua Gong

**Affiliations:** 1 Guelph Research and Development Centre, Agriculture and Agri-Food Canada, Guelph, Ontario, Canada; 2 Next Generation Sequencing Platforms, Clinical Genomics Centre, the UHN/MSH Gene Profiling Facility, Toronto, Ontario, Canada; 3 State Key Laboratory of Food Science and Technology, Nanchang University, Nanchang, Jiangxi, China; 4 Department of Animal and Poultry Science, University of Guelph, Guelph, Ontario, Canada; 5 Department of Animal Science, University of Manitoba, Winnipeg, Manitoba, Canada; 6 Hubei Key Laboratory of Animal Nutrition and Feed Science, Wuhan Polytechnic University, Wuhan, Hubei, China; Universite Paris-Sud, FRANCE

## Abstract

**Background & Aims:**

Butyrate has been shown to potently regulate energy expenditure and lipid metabolism in animals, yet the underlying mechanisms remain to be fully understood. The aim of this study was to investigate the molecular mechanisms of butyrate (in the form of butyrate glycerides, BG)-induced lipid metabolism at the level of gene expression in the jejunum and liver of broilers.

**Methodology/Principal Findings:**

Two animal experiments were included in this study. In Experiment 1, two hundred and forty male broiler chickens were equally allocated into two groups: 1) basal diet (BD), 2) BG diets (BD + BG). Growth performance was compared between treatments for the 41-day trial. In Experiment 2, forty male broiler chickens were equally allocated into two groups. The general experimental design, group and management were the same as described in Experiment 1 except for reduced bird numbers and 21-day duration of the trial. Growth performance, abdominal fat deposition, serum lipid profiles as well as serum and tissue concentrations of key enzymes involved in lipid metabolism were compared between treatments. RNA-seq was employed to identify both differentially expressed genes (DEGs) and treatment specifically expressed genes (TSEGs). Functional clustering of DEGs and TSEGs and signaling pathways associated with lipid metabolism were identified using Ingenuity Pathways Analysis (IPA) and DAVID Bioinformatics Resources 6.7 (DAVID-BR). Quantitative PCR (qPCR) assays were subsequently conducted to further examine the expression of genes in the peroxisome proliferator-activated receptors (PPAR) signaling pathway identified by DAVID-BR. Dietary BG intervention significantly reduced abdominal fat ratio (abdominal fat weight/final body weight) in broilers. The decreased fat deposition in BG-fed chickens was in accordance with serum lipid profiles as well as the level of lipid metabolism-related enzymes in the serum, abdominal adipose, jejunum and liver. RNA-seq analysis indicated that dietary BG intervention induced 79 and 205 characterized DEGs in the jejunum and liver, respectively. In addition, 255 and 165 TSEGs were detected in the liver and jejunum of BG-fed group, while 162 and 211 TSEGs genes were observed in the liver and jejunum of BD-fed birds, respectively. Bioinformatic analysis with both IPA and DAVID-BR further revealed a significant enrichment of DEGs and TSEGs in the biological processes for reducing the synthesis, storage, transportation and secretion of lipids in the jejunum, while those in the liver were for enhancing the oxidation of ingested lipids and fatty acids. In particular, transcriptional regulators of *THRSP* and *EGR-1* as well as several DEGs involved in the PPAR-α signaling pathway were significantly induced by dietary BG intervention for lipid catabolism.

**Conclusions:**

Our results demonstrate that BG reduces body fat deposition via regulation of gene expression, which is involved in the biological events relating to the reduction of synthesis, storage, transportation and secretion, and improvement of oxidation of lipids and fatty acids.

## Introduction

Broilers are highly genetically selected for an enhanced growth rate to serve as a high-quality protein source for humans; however, a higher growth rate is inevitably accompanied by excessive visceral fatness [1–3], which decreases the feed efficiency for protein deposition and economic profits for broiler raisers. In this regard, effective induction of thermogenic processes or control of lipogenesis and subsequent triglyceride synthesis would be promising strategies to control excess fat accumulation in animals. Some biochemical compounds, such as resveratrol and bile acids can profoundly stimulate thermogenic activities and increase energy expenditure [4,5], and therefore decrease energy deposition in adipose tissues in animals. Of particular interest, butyric acid, a short chain fatty acid naturally produced from fiber fermentation in the hind gut of animals, has attracted special interest due to its potential for altering immune response and lipid metabolism in the host. For example, butyric acid and its derivate, namely sodium butyrate and butyrate glycerides, have been reported to effectively induce the immune response by up-regulating gene expression of intestinal host-defense peptides and controlling the proliferation of bacterial pathogens [6–8]. Particularly, butyric acid also significantly reduces the systemically circulating concentrations of total triglycerides and cholesterol by increasing fatty acid oxidation and energy expenditure, thus decreasing the occurrence of diet-induced fat accumulation [9]. In addition, previous report indicated that dietary supplementation of butyric acid significantly increased the carcass yield and breast meat as well as decreased abdominal fat content without inducing statistical differences in body weight and feed intake in broilers [10]. Thus, we hypothesized that butyric acid could regulate the nutrient-redistribution to control fat accumulation and improve protein synthesis.

At the tissue and organ level, lipogenesis occurs essentially in the liver, while the adipose tissue is only a storage tissue [11,12]. Lipogenic activity is much greater in the liver than in adipose tissue [13], contributing 80 to 85% of the fatty acids stored in adipose tissue [14]. The jejunum is also an important organ for the transportation of alimentary fat in the form of lipoproteins to storage sites for triglycerides [15]. Although the potential mechanism through which butyrate induced lipid metabolism in these organs in broilers have not been well documented, it can still be inferred from other animals. For example, in mice, dietary butyrate intervention induces the activation of hepatic adenosine 5’-monophosphate-activated protein kinase (AMPK), which controls fatty acid oxidation and suppresses lipogenic gene expression, thereby decreasing liver fat accumulation as well as increasing insulin sensitivity [16,17]. In adipose tissue, butyrate significantly elevates the expression of peroxisome proliferator-activated receptor-γ coactivator-1α and activities of AMP kinase and p38, thus enhancing lipid catabolism and reducing lipid synthesis in abdominal fat [17]. In addition, the jejunum is also an important organ for nutrient absorption and transportation and may play a role for lipid metabolism. Thus, we speculate that butyric acid may induce regulation of intestinal fat absorption and circulating lipoprotein concentrations and impair lipid transport by inhibiting microsomal triglyceride transfer protein in the jejunum as that in the colon [18,19], although further studies are required to conclude. Therefore, the present study was mainly focused on the expression of genes involved in lipid metabolism in the liver and jejunum.

At the molecular level, chicken lipid metabolism is largely regulated by a group of transcription factors as well as their downstream genes [21]. The transcription factors include early growth response-1 (*EGR-1*), sterol regulatory element-binding protein-2 (*SREBF2*), thyroid hormone responsive (*THRSP*), nuclear receptor subfamily 1, group H, member 3 (*NR1H3*), liver-activated receptor alpha (*LXRA*), and peroxisome proliferator-activated receptors (PPARs) [21–23]. Their downstream genes, such as genes of acyl-coenzyme A oxidase family, acyl-CoA dehydrogenase, long chain (*ACADL*), fatty acid elongase 1 (*ELOVL1*), fatty acid synthase (FASN), were involved in fatty acid synthesis, elongation, and desaturation, while genes of fatty acid binding protein (FABP) family and lipoprotein lipase (*LPL*) were involved in fatty acid transport. In addition, other genes, *e*.*g*. insulin-like growth factor 2 receptor (IGF2R), insulin-like growth factor binding protein (IGFBP) family, and somatostatin receptor 2 (SSTR2), were also involved in lipid metabolism [21]. The expression of these genes was significantly different between genetically fat and lean or high and low feeding-efficiency chickens [20,21,24]. However, how these transcription factors and their downstream genes respond to dietary butyrate intervention in lipid metabolism is still unclear.

In animals, butyric acid could be released from butyrate derivate feed additives, such as sodium butyrate and butyrate glycerides (BG), in the gastrointestinal tract [25]; however, in practice, butyric acid and sodium butyrate could be immediately absorbed by the upper digestive tract [26], therefore limiting the delivery of a sufficient amount of butyric acid to the target tissues or organs for the initiation of its beneficial effects. Butyrate glycerides have no such limitation as they could be released only under the action of lipases in the small intestine [27,28]. Thus, the effectiveness of butyric acid would be improved when it is protected from the absorption in the upper tract. In addition, unlike butyric acid, butyrate glycerides have no offensive odor and are easy to handle [28]. Therefore, the present study sought to investigate the effect of feeding butyrate in the form of a mixture of mono-, di-, and triglycerides on lipid catabolism in broiler chickens, and to identify potential molecular mechanisms underlying the BG-induced effects using RNA-seq technology and two separate bioinformatics analysis systems, namely Ingenuity Pathway Analysis (IPA) and DAVID Bioinformatics Resources 6.7 (DAVID-BR), followed by further examination of selected gene expression with quantitative PCR (qPCR) assays.

## Materials and Methods

### Animals, Experimental Design and Diets

Two animal experiments were included in this study, one was for growth performance and the other was for potential molecular mechanism study relevant to the dietary treatments. The protocol for animal trials was approved by the Animal Care and Use Committee of University of Guelph. The butyrate derivate products used in the current study were mono-butyrin (mono-C4) and a mixture of 30% mono-, 50% di-, and 20% triglycerides of n-butyric acid (Baby C4), commercially available from SILO (Industria Zootecnica, Florence, Italy). The Mono C4 has antimicrobial activity against enteric pathogens [28], while the Baby C4 is beneficial to the development of intestinal epithelium [29]. The dose selection of butyrins was based on previous findings from *in vitro* antimicrobial experiments and a feeding trial with broilers [10,28]. No antibiotics were used throughout the two experiments.

#### Experiment 1

Two hundred and forty 1-day-old male broiler chickens (Ross 308) were allocated equally on the basis of weight of origin into two dietary treatments: 1) basal diet (BD)-fed group; 2) BG diet (BD + BG)-fed group. There were 30 birds per pen with 4 replicate per treatment. The BG referred to two commercial butyrate derivate products, namely Baby C4 and Mono C4. The birds in the BD diet-feed group consumed a commercial diet (Starter, 0 to 20 d) while those in the BG diet-feed group were feed Starter diet containing 3,000 ppm each of Mono C4 and Baby C4 for 0 to 7 d and 3,000 ppm of Mono C4 only for 8 to 20 d. However, the birds in both treatments were feed the same diets (Grower for 21 to 33 d; Finisher for 34 to 40 d) phases. Feed ingredients and feeding program are summarized in S1 Table. All birds were maintained at a brooding temperature of 32°C for 5 d, and then the environmental temperature was gradually reduced to 22°C in keeping with normal brooding practice. The lighting schedule was 23 h/d from d 0 to 4; 18 h/d from d 5 to 18; 20 h/d from d 19 to 28; and 23 h/d from d 29 to 40. Birds were vaccinated against coccidiosis at 1 d of age using gel-spray coccidiosis vaccine (Vetech Laboratories Inc., Guelph, Canada). Feed and water were freely available. Birds were individually weighed prior to feeding in the morning on day 0, 7, 20, 33 and 40. Group feed intakes were recorded weekly. The phase body weight gain, feed intake as well as feed efficiency (ratio of feed intake/body weight gain) were calculated.

#### Experiment 2

Forty 1-d-old male birds were allocated equally into two dietary treatments with 20 birds per treatment. General experimental design, group and management were the same as described in Experiment 1. The lighting schedule was 23 h/d from d 0 to 4; 18 h/d from d 5 to 18; and 20 h/d from d 19 to 20. Group feed intakes were recorded weekly. Birds were individually weighed prior to feeding in the morning on day 0 and 20. The body weight gain was also calculated. All birds appeared healthy and grew well throughout the entire experimental period. On d 20, after weighing, six birds were randomly selected from each treatment and the blood samples were collected from the jugular vein into 5 mL heparin-free vacutainer tubes (Becton Dickinson Vacutainer Systems, Franklin Lakes, NJ, USA). All samples were centrifuged at 750 g for 10 min at 4°C, the supernatant (serum) was immediately collected and placed into test-tubes and stored at -20°C until analysis. After blood collection, the birds were killed by cervical dislocation and used for tissue sampling. Approximately 0.5 g liver was washed with pre-cooled PBS followed by removal of excess blood, and 2 cm middle jejunum were collected followed by removal of digesta as well as connective adipose tissues, and then both tissue samples were placed immediately in RNA-later (Cat #: AM7024, Life Technologies Inc., Burlington, ON, Canada) after cutting into pieces (5 x 5 mm) and frozen at -20°C until further processing for RNA extraction. Approximately 10 g liver, 5 g abdominal fat and 10 cm middle jejunum were also collected, immediately frozen in liquid nitrogen, and stored at -80°C for protein and enzyme analyses. The remaining 14 birds in each treatment were also euthanized, and the whole abdominal fat pads (adipose tissues surrounding the gizzard, bursa of fabricius, cloaca and adjacent muscles) were removed and weighed following the procedures as reported previously [30].

### Serum Biochemical Analyses

Serum concentrations of glucose, triglycerides, total cholesterol, low-density lipoprotein-cholesterol (LDL-C) and high-density lipoprotein-cholesterol (HDL-C) were determined using an Automatic Biochemical Analyser (Beckman, Miami, FL, USA) with corresponding kits (glucose, Cat #: ab65333; triglycerides, Cat #: ab65336; total cholesterol, Cat #: ab65359; HDL-C and LDL-C, Cat #: ab65390) commercially available from Abcam Inc. (Cambridge, MA, USA). Serum concentrations of total protein, acyl-coenzyme A oxidase β (ACACB), FASN and LPL were measured using an ELX800 Absorbance Microplate Reader (Bio-Tec Instruments Inc., Winooski, VT, USA) with corresponding ELISA kits according to the manufacturers’ instructions.

### Tissue Levels of Selected Enzyme Analyses

The tissue levels of selected enzymes associated with lipid metabolism were determined according to previous procedures with minor modifications [31]. Briefly, 1 g tissue sample was pulverized in liquid nitrogen, and then 100 mg pulverized sample was collected and placed into 1 mL ice-cold lysis buffer (Cat #: T2327, Life Technologies Inc.) and homogenized in an ice-water bath. The lysis buffer contained 1% protease inhibitor cocktail (Cat #: P8340, Sigma, St Louis, MO, USA). Following homogenization, samples were centrifuged at 10,000 g for 5 min at 4°C. After removal of the fat cake, the resulting supernatant was collected, and the pellet was re-suspended in 1 mL lysis buffer to repeat the process of homogenization and centrifugation as described above. The two collections of supernatant were combined and used for enzyme assays. The concentrations of total protein, ACACB, FASN and LPL in the supernatant were determined with the same kits and instruments as described for serum analyses.

### RNA Extraction

Total RNA of liver and jejunum samples was extracted using Ambion mirVana^™^ miRNA isolation kit (Cat #: AM1560, Life Technologies Inc.) in accordance with the manufacturer’s instructions. The RNA integrity and concentration were evaluated using an Agilent 2100 Bio-analyzer (Agilent Technologies, Palo Alto, CA, USA) and then treated with Ambion DNase I (Cat #: 18068–015, Life Technologies Inc.) following the manufacturers’ instructions. The average RNA integrity number (RIN) was 9.5.

### Transcriptome Sequencing

Equal amounts of total RNA (2 μg) from three individual birds were pooled within each treatment as described previously with minor modification [32]. The mRNA-seq libraries were constructed from 6 μg pooled RNA using the Illumina mRNA-seq sample preparation kit (Cat #: RS-930-1001, Illumina Inc., San Diego, CA, USA). For each treatment and each organ, two sequencing libraries were constructed from the pooled RNA samples. The rationale for pooling RNA samples from individual samples is that RNA pooling is cost-effective and can basically obtain genome-wide information about potentially functionally relevant variations [33,34]. In addition, one purpose of the RNA-seq analysis in the current study was for comprehensive screening, and some identified genes were also examined for their expression by qPCR assays. Each library was loaded into a single lane of the Illumina Genome Analyzer II system (Illumina Inc.) and subjected to paired-end sequencing performed with the corresponding kits (TruSeq PE Cluster kit v5-CS-GA, Cat #: PE-203-5001 and TruSeq SBS Kit v5-GA, Cat #: FC-104-5001, Illumina Inc.).

### RNA-seq Data Process and Analysis

After obtaining the short reads using Bustard (Illumina Pipeline version 1.3), a series of quality checks were performed. Briefly, reads were quality-filtered using the standard Illumina pipeline and the reads that passed filtering were then processed and aligned to the *Gallus gallus* NCBI build 3.1 reference genome, provided through the Illumina iGenomes project, using TopHat v2.0.8 [35]. TopHat incorporates the Bowtie v2.0.6 algorithm to perform the alignment [36]. The resulting BAM alignment files were then converted to SAM files using SAM Tools and a table of read counts was generated using HTSeq-count [37,38], part of the HTSeq python package (http://www-huber.embl.de/users/anders/HTSeq/doc/overview.html), for input into DESeq. The DESeq v1.10.1 implemented in R v2.15.3 was employed to normalize the read counts and detect genes expressed differentially between treatment groups [32,39]. The two sided *P*-value was corrected using the false discovery rate (FDR) adjustment to account for multiple hypothesis testing procedures [40]. For genes expressed in both groups, a cutoff with the FDR adjusted *P*-value ≤v0.05 using the Benjamini and Hochberg procedure was considered to be statistically significant. To ensure biological relevance, a condition of absolute value of Log_2_ fold-change ≥ 1.0 (fold change threshold ± 2) was added on top of FDR-adjusted *P*-value (≤ 0.05). Thus, genes with FDR-adjusted *P*-value (≤ 0.05) and absolute value of Log_2_ fold-change ≥ 1.0 were considered to be differentially expressed in the current study [33, 40, 41]. The genes with a normalized count number ≥ 5 were considered to be treatment-specifically expressed. For the DEGs, the gene abbreviations with their NCBI gene IDs, corresponding adjusted *P*-values and fold changes were uploaded to the IPA and DAVID-BR online bioinformatics analysis systems for biological functional annotation and canonical pathways analyses. For treatment specifically expressed genes (TSEGs) that were detected in either BG-fed or BD-fed chickens, the gene abbreviations with their NCBI gene IDs were uploaded to the same bioinformatical analysis system for further analysis. A *P*-value determining the probability that each biological function and/or canonical pathway is due to chance alone was determined by Fischer’s exact test [41,42].

### Reverse Transcription and Quantitative PCR Assay

The DEGs involved in PPAR*-α* signaling pathway as identified by the DAVID-BR analysis were also examined for their expression by qPCR analysis. These genes include Fatty acid binding protein 4 (*FABP-4*), *LPL*, matrix metalloproteinase-1 (*MMP-1*) and perilipin-1 (*PLIN-1*) in the jejunum and cytochrome P450 family 8 subfamily B polypeptide 1 (*CYP8B1*), *FABP-2*, *LPL* and *MMP-1* in the liver, respectively. Jejunum and liver samples from six individual birds of each group were used. Two micrograms of total RNA was used for cDNA synthesis using a High Capacity cDNA Reverse transcription Kit (Cat #: 4368814, Applied Biosystems, Foster City, CA, USA) with RNase inhibitor (Cat #: N8080119, Applied Biosystems) according to the manufacturer’s instructions. The qPCR assays were performed using a S1000 Thermocycler (1852148, Bio-Rad Laboratories, Hercules, California) with iTaq^™^ Universal SYBR^**®**^ Green Supermix (1725121, Bio-Rad Laboratories, Hercules, California). Briefly, cDNA was diluted 10-fold, and 1 μL of each diluted sample was added to a 25- μL volume reaction, also containing 12.5 μL quantitative PCR Master Mix, 1 μL of each forward and reverse primers of the target genes (10 pmol/uL) and 9.5 μL nuclease-free water (Cat #: AM9937, Life Technologies Inc.). The amplification program started at 94°C for 3 min followed by 40 cycles of 94°C for 30 s, the gene-specific annealing temperature for 30 s and extension at 72°C for 30 s. Fluorescence measurements were collected after each annealing step. The PCR primers targeting the genes were designed using NCBI primer blast (http://www.ncbi.nlm.nih.gov/tools/primer-blast/) with corresponding mRNA sequences (S2 Table). The gallinaceous *β*-actin gene was used as an internal reference to normalise target gene transcript levels, as its stability has been verified in chickens previously [43]. In addition, its Ct values with the jejunum and liver samples were also shown to be stable in the present study. Negative controls were created by replacing cDNA with water. The relative abundance of target gene was analyzed using the 2^-ΔΔCt^ method [44]. The values derived from 2^-ΔΔCt^ represent fold changes of samples in the abundance relative to the control group. The identity of each amplicons was confirmed by sequencing at Laboratory Services of University of Guelph (Guelph, ON, Canada).

### Statistical Analysis

Since the purpose of the present study was to evaluate the effect of butyric acid as whole on young broilers from a nutritional perspective for chicken production, the statistical analysis of data on growth performance, fat deposition, serum concentrations of selected metabolites and enzymes, and expression of selected genes involved in lipid metabolic process was performed using the TTEST procedure of SAS (version 9.3, SAS Institute Inc., Cary, NC, USA) with repeated unpaired observations. A *P* < 0.05 value was taken to indicate statistical significance between treatments.

## Results

### Growth Performance, Fat Deposition and Serum Glucose and Lipid Profiles

In Experiment 1, the addition of BG did not affect the body weight gain and feed intake; however, a better feed efficiency (*P* < 0.05) was observed in the BG-fed chickens from d 8 to 20 of the trial compared with the control group. In addition, a reduction of abdominal fat weight in BG-fed chickens was observed, although no effort was made for statistical analysis. Experiment 2 basically reproduced the results obtained in Experiment 1. Dietary addition of BG improved the final body weight and body weight gain (*P* = 0.01) of young birds while decreased the ratio of abdominal fat weight/final body weight (*P* = 0.01) compared with the BD-fed group (Table 1). In addition, dietary BG intervention did not affect serum concentrations of glucose and LDL-C (*P* > 0.05), but increased (*P* < 0.01) the serum concentration of total protein as well as decreased (*P* < 0.05) total cholesterol, triglycerides and HDL-C when compared with the control group (Table 2).

**Table 1:** Effect of butyrate glycerides on growth performance, fat deposition and small intestinal development in broilers.

Trials		Treatment			
Experiment 1	Item	BD-fed group	BG-fed group	*P*-value	Pooled SEM
	IBW (g/bird)	42.0	41.5	0.25	0.01
	FBW (g/bird)	2934	3001	0.15	8.72
	**FI (g/bird)**				
	0 ~ 7 d	143.3	141.9	0.22	0.47
	8 ~ 20 d	917.8	864.8	0.16	2.87
	21 ~ 33 d	2180	2244	0.43	5.99
	34 ~ 40 d	1529	1578	0.24	5.62
	0 ~ 40 d	4770	4829	0.76	12.51
	**BWG (g/bird)**				
	0 ~ 7 d	126.0	121.0	0.13	0.04
	8 ~ 20 d	623.8	660.3	0.59	0.93
	21 ~ 33 d	1349.2	1382.5	0.57	2.67
	34 ~ 40	793	796	0.82	5.69
	0 ~ 40 d	2892	2960	0.76	7.66
	**F/G (g/g)**				
	0~7 d	1.14	1.16	0.12	0.00
	8~20 d	1.47 ^a^	1.31	0.01	0.00
	21~33d	1.61	1.62	0.15	0.00
	34~40 d	1.93	1.98	0.26	0.00
	0~40 d	1.65	1.63	0.17	0.00
Experiment 2	IBW (g/bird)	41.0	41.1	0.49	0.07
	FBW (g/bird)	862.3	992.7	0.01	0.37
	FI (g/bird)	1161.1	1206.7	0.14	6.51
	BWG (g/bird)	821.3	951.6	0.01	0.05
	AFW (g/bird)	13.1	13.1	0.24	0.02
	AFW/FBW (%)	1.51	1.32	0.01	0.00

n = 4 pens (30 birds per pen) for Experiment 1; n = 20 birds for the IBW and FI and n = 14 birds for the FBW, BWG, AFW and AFW/FBW in Experiment 2. AFW, Abdominal fat weight; BD, Basal diet; BG, Butyrate glycerides; FBW, Final body weight; FI, Feed intake; IBW, Initial body weight; BWG, Body weight gain.

**Table 2:** Effect of butyrate glycerides on concentrations of serum metabolites as well as total protein and selected key enzymes in selected tissues in broilers.

Tissue	Item	BD-fed group	BG-fed group	*P*-value	Pooled SEM
Serum	Glucose (mmol/L)	14.0	13.3	0.10	0.28
	Total cholesterol (mmol/L)	4.0	3.6	0.04	0.09
	Triglycerides (mmol/L)	1.21	1.0	0.04	0.05
	HDL-C (mmol/L)	2.9	2.6	0.03	0.08
	LDL-C (mmol/L)	0.9	0.8	0.07	0.08
	Total protein (g/L)	18.9	22.8	0.00	0.24
	ACACB (mg/L)	65.2	47.3	0.01	1.88
	FASN (mg/L)	5.1	2.3	0.00	0.19
	LPL (ng/L)	93.8	88.4	0.08	0.87
Jejunum	Total protein (mg.g^-1^ tissue)	227.1	281.1	0.00	0.36
	ACACB (μg.g^-1^ protein)	49.1	40.7	0.06	0.31
	FASN (μg.g^-1^ protein)	1.5	2.0	0.07	0.59
	LPL (μg.g^-1^ protein)	106.4	88.6	0.01	0.08
Liver	Total protein (mg.g^-1^ tissue)	352.1	311.8	0.09	0.24
	ACACB (μg.g^-1^ protein)	13.5	8.5	0.00	1.82
	FASN (μg.g^-1^ protein)	37.4	28.4	0.00	0.09
	LPL (μg.g^-1^ protein)	49.5	39.	0.02	0.77
Abdominal adipose	Total protein (mg.g^-1^ tissue)	27.8	33.8	0.14	1.88
	ACACB (μg.g^-1^ protein)	2.9	2.1	0.06	0.19
	FASN (μg.g^-1^ protein)	4.2	1.7	0.00	0.57
	LPL (μg.g^-1^ protein)	10.4	7.1	0.02	0.86

Notes: agents and kit information for the assays. Total protein (Cat #: 22660, Thermo Scientific Int., Dockford, IL, USA), ACACB (Cat #: E12A0639, Shanghai Blue-Gene Biotech Co., Ltd., Shanghai, China), FASN (Cat #: SEC470Ga, Uscn Life Science Inc., Wuhan, Hubei, China); LPL (Cat #: CSB-E13395C, Cusabio Biotech Co., Ltd., Wuhan, Hubei, China). n = 6 birds. ACACB: acetyl coenzyme A carboxylase β; BD: basal diet; BG: butyrate glycerides; FASN: fatty acid synthase; HDL-C: high-density lipoprotein-cholesterol; LDL-C: low-density lipoprotein-cholesterol; LPL: lipoprotein lipase.

### Serum and Tissue Enzymes Associated with Lipid Metabolism

Dietary BG intervention significantly decreased the serum concentrations of ACACB and FASN (*P* < 0.01), jejunum level of LPL (*P* < 0.01), liver levels of ACACB and FASN (*P* < 0.01) and LPL (*P* < 0.05) as well as abdominal adipose levels of FASN (*P* < 0.001) and LPL (*P* < 0.05), while increasing the jejunum level of total protein (*P* < 0.01) when compared with the BD-fed birds (Table 2).

### Mapped Reads and Detection of Genes and Transcripts

Table 3 shows sequence reads generated by the Illumina Genome Analyzer II system from the jejunum and liver samples, which ranged from the average of 58,633,092 to 63,788,828 reads. Among these reads, 66.4% in the liver and 66.1% in the jejunum in BD-fed birds and 68.5% in the liver and 72.6% in the jejunum in BG-fed chickens were mapped. Statistical analysis indicated that 210 and 87 genes in the liver and jejunum were expressed differentially (*P* < 0.05); however, only 205 and 79 genes were characterized, respectively. Among the characterized genes, 118 genes were up-regulated and 77 down-regulated in the liver (S3 Table), and 25 genes were up-regulated and 54 down-regulated in the jejunum, respectively (S4 Table). In addition to the differentially expressed genes, there were 255 and 165 genes in the liver and jejunum, whose expression was detected only in the BG-fed chickens. Similarly, there were 162 and 211 genes whose expression was only detectable in the liver and jejunum of BD-fed birds, respectively. These genes are designated as treatment specifically expressed genes (TSEGs).

**Table 3:** RNA sequencing profiles.

Item	Jejunum				Liver			
Treatment	BD-fed group		BG-fed group		BD-fed group		BG-fed group	
Library	1	2	3	4	5	6	7	8
Total reads	61,238,182	66,339,474	55,424,390	61,841,794	70,522,886	59,034,130	62,688,832	54,904,032
Mapped reads	43,666,975	40,687,936	41,362,156	43,714,988	48,424,723	37,642,625	42,839,605	37,722,080

Notes: BD, Basal diet; BG, Butyrate glycerides.

### Biological Function Clustering

**Fig 1:**
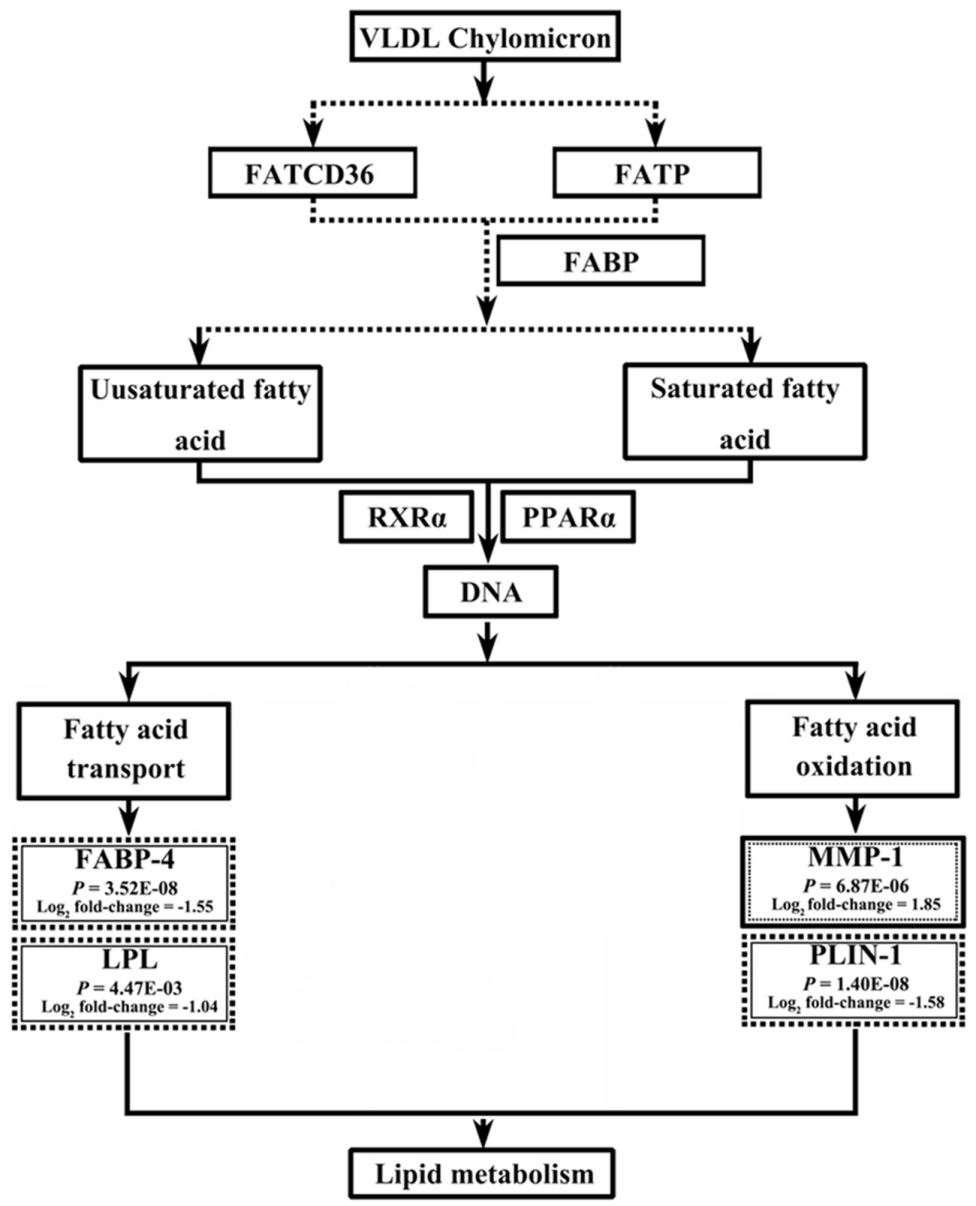
Influence of butyrate glycerides on the genes downstream the PPAR signaling pathway in the jejunum of broilers. The genes in the text box with dotted outer line and full inner line are down-regulated, while those in the text box with full outer line and dotted inner line are up-regulated compared to the control group. All genes showing a significant difference in expression had the cut-off of *P-*value ≤ 0.05 and absolute value of Log_2_ fold change ≥ 1. This figure was generated based on the results of DAVID-BR analysis.

**Fig 2:**
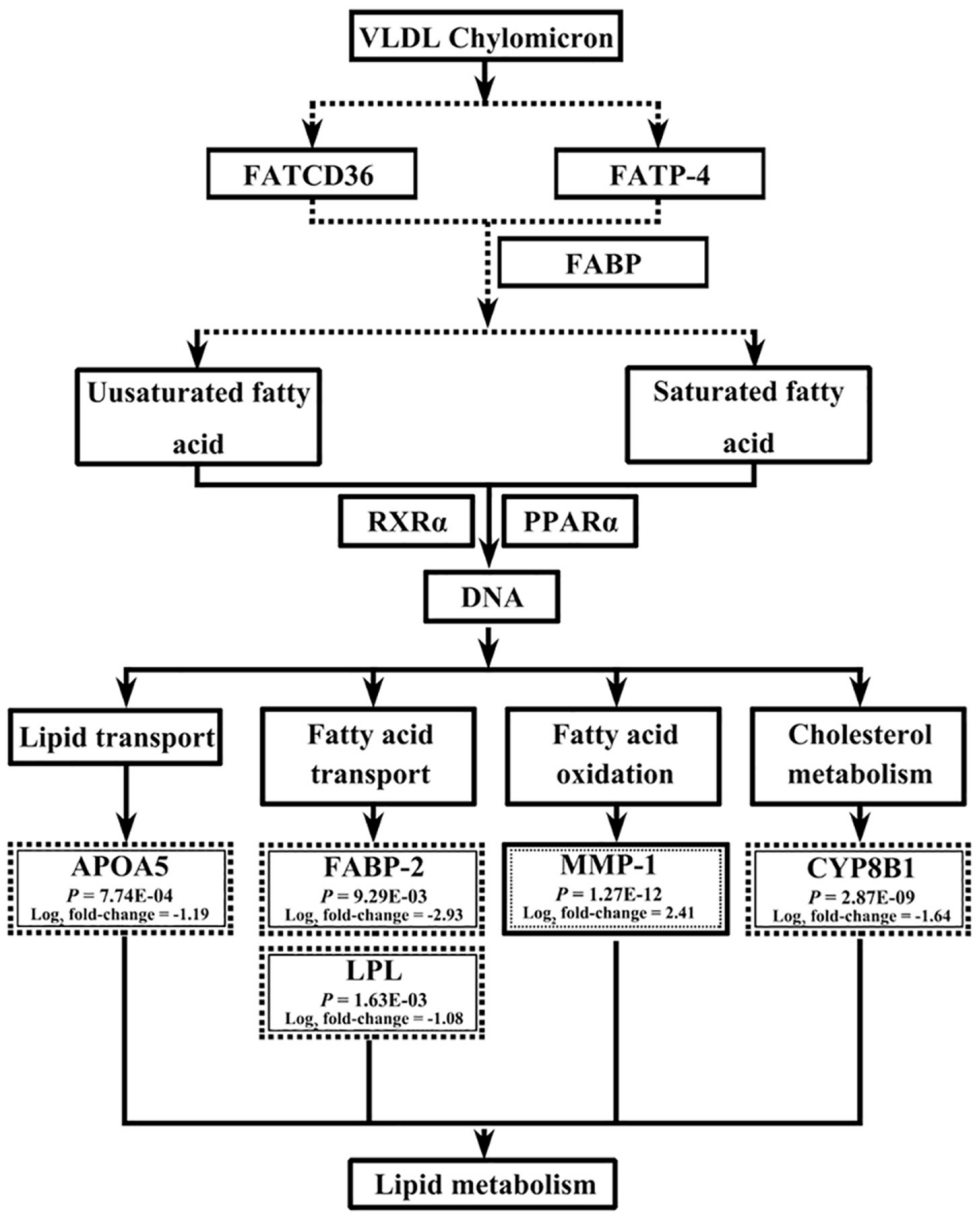
Influence of butyrate glycerides on the genes downstream the PPAR signaling pathway in the liver of broilers. The genes in the text box with dotted outer line and full inner line are down-regulated, while those in the text box with full outer line and dotted inner line are up-regulated compared to the control group. All genes showing a significant difference in expression had the cut-offs of *P-*value ≤ 0.05 and absolute value of Log_2_ fold change ≥ 1. This figure was generated based on the results of DAVID-BR analysis.

RNA-seq and bioinformatics analyses revealed that dietary BG intervention induced a wide range of biological events in both the liver and jejunum, including cell signaling, cellular compromise and inflammatory response, cell morphology, cycle, death, and survival, molecular transport, carbohydrate metabolism and energy production, and lipid metabolism. As indicated in S5 and S6 Tables that were generated from the IPA analysis, lipid metabolism and inflammatory/immunological disease were among the top associated network functions. The lipid metabolism was largely enriched with both DEGs and TSEGs. For example, the core analysis with IPA showed that several networks involved in lipid metabolism were significantly enriched with the DEGs in the jejunum (score = 84) and liver (score = 5) (S5 Table) as well as with the TSEGs in the liver of BD-diet fed birds (score = 54 and 10), respectively (S6 Table). In addition, the major IPA categories under the top biological functions represented by the DEGs include “Diseases and disorders, molecular and cellular functions, as well as physiological system development and function”. Several subcategories under the IPA category “Molecular and cellular functions” revealed the most prevalent biological events including lipid metabolism (20 genes in the jejunum and 6 genes in the liver) as well as other process (Table 4). Moreover, the major biological function significantly enriched with the TSEGs under the IPA category “Molecular and Cellular Functions” was lipid metabolism (10 genes) in the jejunum of BD-fed birds as well as other biological processes in the jejunum and liver of BG and BD-fed birds (Table 5). Further analysis with IPA revealed that 23 DEGs and 29 TSEGs were mainly involved in a number of specific biological events covering different aspects of lipid metabolism in the jejunum and liver of broilers (S7 and S8 Tables). In particular, the expression of early growth response1 (*EGR1*) was significantly increased in the liver (S3 Table), while that of *THRSP* was significantly decreased in the jejunum in BG-fed birds (S4 Table). The signaling pathway analysis with DAVID-BR reveled a significant enrichment of DEGs involved in the PPAR-α signaling pathway in the jejunum (*P* = 2.20E-03) and liver (*P* = 7.70E-03) (Table 6). In the jejunum, *FABP-4*, *LPL* and *PLIN-1* genes were down-regulated and MMP-1 gene was up-regulated in the PPAR-*α* signaling pathway (Fig 1). In the liver, *APOA5*, *FABP-2*, *CYP8B1* and *LPL* genes were down-regulated and MMP-1 was up-regulated in the same signaling pathway (Fig 2). These genes were more specifically involved in lipid metabolism related processes, *e*.*g*. fatty acid transport (*FABP4* and *LPL*) and fatty acid oxidation (*MMP-1* and *PLIN-1*) in the jejunum and fatty acid synthesis (*ACACB*) in the liver.

**Table 4:** Physiological functions enriched with DEGs in response to butyrate glycerides treatment in broilers^a^.

Tissue	Physiological functions	*P*-value	Number of molecular
Jejunum	Lipid metabolism	1.25E-04 ~ 3.10E-02	20
	Molecular transport	1.25E-04 ~ 3.73E-02	28
	Small Molecule Biochemistry	1.25E-04 ~ 3.10E-02	24
	Drug Metabolism	8.92E-04 ~ 3.10E-02	9
	Vitamin and Mineral Metabolism	8.92E-04 ~ 3.73E-02	12
Liver	Lipid metabolism	1.35E-05 ~ 4.40E-02	6
	Small molecule Biochemistry	1.35E-05 ~ 4.78E-02	17
	Cellular assembly and organization	8.07E-05 ~ 4.04E-02	7
	Cell-to-cell signaling and interaction	8.71E-04 ~ 4.40E-02	17
	Cellular growth and proliferation	8.71E-04 ~ 4.76E02	12

^a^ Determined by IPA analysis; n = 2, each sample was a combined sample from three chickens.

**Table 5:** Physiological functions enriched with treatment specifically expressed genes in broilers^a^.

Treatment	Tissue	Physiological functions	*P*-value	Number of molecular
BG-fed group	Jejunum	Molecular transport	5.69E-05 ~ 4.97E-02	19
		Cell signaling	2.33E-03 ~ 2.35E-02	6
		Vitamin and mineral metabolism	2.33E-03 ~ 2.35E-02	5
		Carbohydrate metabolism	5.53E-03 ~ 4.62E-02	6
		Small molecule biochemistry	5.53E-03 ~ 4.65E-02	20
	Liver	Cell-to-cell signaling and interaction	9.90E-05 ~ 4.61E-02	49
		Cell signaling	2.53E-04 ~ 4.47E-02	11
		Cell death and survival	8.05E-04 ~ 2.33E-02	9
		Cell morphology	1.09E-03 ~ 4.61E-02	23
		Nucleic acid metabolism	1.68E-03 ~ 4.47E-02	9
BD-fed group	Jejunum	Lipid metabolism	5.36E-05 ~ 3.18E-02	10
		Molecular transport	5.36E-05 ~ 2.92E-02	19
		Small molecule biochemistry	5.36E-05 ~ 3.18E-02	17
		Cell-to-cell signaling and interaction	1.02E-04 ~ 3.64E-02	20
		Cellular compromise	5.28E-04 ~ 2.92E-02	10
	Liver	Molecular transport	2.63E-04 ~ 4.96E-02	24
		Small molecule biochemistry	1.59E-03 ~ 4.96E-02	14
		Cell-to-cell signaling and interaction	2.08E-03 ~ 4.38E-02	15
		Cellular assembly and organization	2.08E-03 ~ 3.74E-02	8
		Cellular function and maintenance	2.08E-03 ~ 4.96E-02	10

^a^ Determined by IPA analysis; n = 2, each sample was a combined sample from three chickens.

BD, Basal diet; BG, Butyrate glycerides.

**Table 6:** Associated KEGG pathways in response to butyrate glycerides treatment in broilers^a^.

Tissue	Associated pathways	Number of genes	*P*-value
Jejunum	PPAR signaling pathway	4	2.20E-03
	Nitrogen metabolism	2	9.50E-02
Liver	Cell cycle	8	6.50E-04
	PPAR signaling pathway	5	7.70E-03
	Oocyte	6	9.10E-03
	Progesterone-mediated oocyte maturation	5	1.70E-02

^a^ Determined by DAVID-BR analysis; n = 2, each sample was a combined sample from three chickens.

### Examination of DEGs Involved in PPAR-*α* Signaling Pathway with qPCR

The expression of DEGs involved in PPAR-*α* signaling pathway in the jejunum or liver, as identified by DAVID-BR, were further examined for their expression with qPCR assays. All the genes in the tissues (either liver or jejunum) showed the same direction in their expression (either up-regulated or down-regulated, *P* < 0.01) in qPCR assays as in the RNAseq analysis, as the response to dietary BG-intervention, except that the up-regulation of *MMP-1* in the liver was not significant (*P* = 0.07) in the qPCR assay (Table 7). Thus, the qPCR data basically confirmed the results from the RNA-seq analysis on the selected genes.

**Table 7:** Comparison in the results of RNA-seq and qPCR analyses of selected genes involved in PPAR-α signalling pathway in respond to butyrate glycerides treatment in broilers^a^.

Tissue	Gene	RNA-seq		qPCR	
		Log_2_ fold-change	*P* value	Log_2_ fold-change	*P* value
Jejunum	FABP-4	-1.55	< 0.001	-1.12	< 0.01
	LPL	-1.04	< 0.001	-1.00	< 0.001
	MMP-1	1.85	< 0.001	1.65	< 0.001
	PLIN-1	-1.58	< 0.001	-0.62	< 0.01
Liver	APOA5	-1.19	< 0.001	ND	ND
	CYP8B1	-1.64	< 0.001	-1.51	< 0.001
	FABP-2	-2.93	< 0.001	-1.89	< 0.001
	LPL	-1.08	< 0.001	-1.25	< 0.001
	MMP-1	2.41	< 0.001	0.78	0.07

*β –actin*: actin, beta (HGNC: 132); *APOA5*: apolipoprotein A-V (HGNC: 17288); *CYP8B1*: cytochrome P450, family 8, subfamily B, polypeptide 1 (HGNC: 2653); *FABP2*: fatty acid binding protein 2, intestinal (HGNC: 3556); *FABP4*: fatty acid binding protein 4, adipocyte (HGNC: 3559); *LPL*: lipoprotein lipase (HGNC: 6677); *MMP1*: matrix metallopeptidase 1 (HGNC: 7155); *PLIN1*: perilipin 1 (HGNC:9076).

^a^ n = 6 chickens;

ND: not determined.

## Discussion

It is generally accepted that abnormal fat deposition, principally stored as triglycerides in the adipose tissue, mainly arises when energy intake exceeds energy expenditure [45]. In some cases, excess triacylglycerol can also be deposited in other organs, such as the kidney, skeletal muscle and blood vessels, as the result of hypertrophy and hyperplasia of adipocytes [46]. A notable result from the current study was the significant decrease in the ratio of abdominal fat weight to the final body weight without any differences in feed intake, indicating a decrease of relative fat deposition in BG-fed chickens. Given that butyrate can be released from BG through lipases in the small intestine [27], we speculate that the decreased fat deposition could be achieved through the redistribution of ingested energy induced by butyrate acid. However, much remains to be learned about how butyrate (or BG) regulates the balance between synthesis, uptake, and transport of fatty acids.

Fat deposition is a complex process, which is associated with lipogenic and lipolytic capacity, as well as fatty acid transport and utilization in the liver and adipose tissues [47]. Lipogenesis is a process by which simple sugars such as glucose are converted to fatty acids [48], and is regulated by a wide array of interdependent factors, including nutrients, hormones, nuclear transcription factors and lipogenic enzymes [49,50]. In this regard, the circulating glucose and lipid profiles may reflect the effects of dietary treatments on lipid metabolism to a certain extent. In the current study, although dietary BG intervention did not affect serum concentrations of glucose and LDL-C, the concentrations of total cholesterol, triglycerides, and HDL-C were significantly lower in BG-fed birds, indicating that the adipogenesis processes were attenuated. This speculation was further supported by the fact that the levels of ACACB, FASN and LPL in the selected tissues (jejunum, liver and abdominal adipose), which are closely associated with lipid metabolism, were also affected. The ACACB, which catalyzes the carboxylation of acetyl-CoA to malonyl-CoA, is a key rate limiting enzyme in the *de novo* pathway of fatty acid synthesis [51,52]. The FASN, which catalyzes the entire pathway of palmitate synthesis from malonyl-CoA, is a key determinant of the maximal capacity of a tissue to synthesize fatty acids by the *de novo* pathway [53,54]. In the current study, the significant decrease in ACACB and FASN at the protein level detected in various tissues and serum may have contributed to the reduction of lipogenesis in BG-fed broilers, and therefore decreased the accumulation of fat mass in the whole body. However, the RNA-seq analysis did not detect the difference of ACACB in the jejunum and of FASN in both jejunum and liver between treatments. This discrepancy could be due to the lower correlation between RNA and protein expression profiles as reported previously [55–57]. The LPL is the major enzyme responsible for hydrolysis of triglycerides in chylomicrons and very low-density lipoprotein (VLDL) to provide free fatty acids for tissue utilization or storage as well as to promote the cellular uptake of chylomicron remnants, cholesterol-rich lipoproteins, and free fatty acids [58,59]. In addition, it is responsible for binding and transportation of fatty acid in the blood stream and intestine [60]. The lower concentrations of LPL in the liver, jejunum and abdominal adipose as well as its expression in both jejunum and liver detected by both DESeq v1.10.1 implemented in R v2.15.3 analysis and qPCR analysis suggest that the hydrolysis of triglycerides in chylomicrons, VLDL and fatty acid as well as the lipid transportation in the jejunum were reduced in BG-fed chickens. Taken together, these data indicate that dietary BG reduces the capacity of liver and adipose tissues for *de novo* synthesis of fatty acids and the hydrolysis of triglycerides that can release fatty acids, as well as the transportation of fatty acids from the jejunum to blood stream, thus leading to the reduction of body fat accumulation.

The major finding from the current study was the observation that the expression of *THRSP*, *EGR-1*, and several downstream genes involved in the PPAR-α signaling pathway was significantly affected by dietary BG intervention. The THRSP is a key transcriptional regulator of lipogenesis and adipo-genesis [20], which was shown highly expressed in genetically fat birds as identified with microarray and RNA-seq analysis [21,61]. However, in the current study, its expression was down-regulated in the jejunum, as indicated by the RNA-seq analysis, indicating that the lipogenesis and adipo-genesis was decreased in the intestinal region of BG-fed birds [62]. The *EGR-1* is a pleiotropic transcription factor. Up-regulation of its expression would induce the expression of insulin like growth factor 2 (*IGF-2*), platelet derived growth factors (*PDGF*) and fibroblast growth factors (*FGF*) [63,64], thereby promoting lipid oxidation, triglyceride clearance and ketogenesis [65]. In the current study, the expression of *EGR-1* was up-regulated in the liver of BG-fed birds, indicating an improvement of lipid oxidation, triglyceride clearance and ketogenesis. It has been well known that the PPAR-α signaling pathway can profoundly affect lipid metabolism [66,67]. In the present study, the expression of several downstream genes involved in lipid metabolism of PPAR-α signaling pathway was significantly affected by dietary BG intervention, including *FABP-4*, *LPL*, *MMP-1* and *PLIN-1* in the jejunum and *APOA5*, *CYP8B1*, *FABP-2*, LPL and *MMP-1* in the liver, which has mostly been confirmed by both RNA-seq and qPCR analysis. These genes mainly play a role in lipogenesis, fatty acid transport and oxidation, lipid transport or cholesterol metabolism. For example, proteins encoded by FABP family genes are responsible for fatty acid transport and metabolism [68,69] and the lower transcription level of *FABP4* and *FABP2* suggests that the transportation of fatty acid was suppressed in response to dietary BG-intervention in the jejunum and liver. The *LPL* encoded protein activity has been shown to be closely related to adipose mass in chickens [70]. In this regard, the lower transcription level of *LPL* in the jejunum and liver was consistent with its lower concentration in corresponding tissues, indicating lower fat accumulation. The PLIN-1 protein is a fundamental regulator of lipolytic activity, which facilitates the recruitment of lipases and other regulatory proteins to lipid droplet surfaces [71,72]. Loss of PLIN-1 activity significantly reduces intracellular lipid levels in adipocytes and cause leanness in mice [73]. Thus, it can be speculated that the down-regulation of PLIN-1 would result in lower lipid levels in the jejunum of BG-fed birds. The *APOA5* encoded protein is an important modulator exclusively relating to lipoproteins, such as High-density lipoprotein (HDL), VLDL and chylomicronsm [74]. Down-regulation of its expression observed in the current study would induce an increase of the levels HDL, VLDL and chylomicronsm and ameliorate corresponding lipids transportation and clearance in the liver of BG-fed birds.

In addition to the DEGs that are either transcriptional regulators or involved in the PPARs signaling pathway, there were some DEGs with different functions, which actively responded to dietary BG-induced lipid metabolism, including cytochrome P450 2C9 (*CYP2C9*), alcohol dehydrogenase 1C (*ADH1C*) and recombination activating gene 2 (*RAG2*). The CYP2C9 is an important cytochrome P450 enzyme with a major role in the oxidation of both xenobiotic and endogenous compounds, which also catalyzes many reactions involved in drug metabolism [75,76]. The up-regulation of its expression observed in the jejunum in the present study suggests attenuation in the synthesis of cholesterol, steroids and other lipids in the intestinal region. The ADH1C is a member of alcohol dehydrogenase [77]. In the small intestine, fatty acids are a substrate for alcohol dehydrogenase, which generates an oxo-fatty acid followed by generation of the corresponding dicarboxylic acid via the action of aldehyde dehydrogenases (*ALDH*) [78,79]. Up-regulation of the expression of *ADH1C* in the current study would stimulate the oxidation fatty acid and thereby decrease the absorption of ingested fatty acid into systemic circulation. The *RAG-2* induces V(D)J-specific recombination when transfected into fibroblasts where adipogenesis is stimulated by PPAR-*γ* [80]. In this regard, it can be speculated that the down-regulation of *RAG-2* expression could inhibit the PPAR-*γ* activity indirectly, and therefore decrease adipogenesis in the jejunum of BG-fed birds. Taken together, these data suggest that dietary BD intervention induced multiple factors or processes to reduce fat deposition in broilers.

Some TSEGs may have also contributed to lipid catabolism in the liver and jejunum in BG-fed birds. Among these genes, cholesterol 25-hydroxylase (*CH25H*) and phospholipase A2-group II A (*PLA2G2A*) had a higher normalized average read number in the liver and jejunum, respectively, in the BG-fed birds. The *CH25H* indirectly regulates the activation of sterol regulatory element binding proteins (SREBPs) through hydroxylation of cholesterol to 25-hydroxycholesterol [81]. The SREBPs is a transcription factor that regulates genes involved in the synthesis of cholesterol and other lipids in animal cells [82], while the *PLA2G2A* preferentially hydrolyses per-oxidized fatty acid esters in phospholipid membranes [83,84], which also hydrolyses other lipids, such as arachidonic acid and lysophosphatidylcholine. Therefore, in the present study the BG-specifically associated effect of *CH25H* may have indirectly inhibited the synthesis of cholesterol and lipids in the liver. Similarly, the BG-specifically associated effect of *PLA2G2A* may have stimulated lipids hydrolysis, leading to a decrease in the net absorption of dietary lipids in the small intestine of BG-fed chickens. To better understand the potential mechanism of BG-induced lipid catabolism, the genes specifically expressed in the BD-fed group were also taken into account. In particular, the steroidogenic acute regulatory protein (*StAR*) in the BD-fed group also had a higher normalized average read number. The *StAR* plays a critical role in steroidogenesis by enhancing the delivery of substrate cholesterol from the outer mitochondrial membrane to the cholesterol side chain cleavage enzyme system on the inner membrane [85]. The up-regulation of *StAR* in the jejunum observed in the present study suggests an increase in the transport of cholesterol in the BD-fed chickens. Collectively, the results discussed above indicate that the ingested energy can largely be metabolized within the liver and jejunum through different biological events that were induced by the BG treatment.

There is also a possibility that the high redox state and energy expenditure through highly oxidative respiratory induced by dietary BG-intervention may have indirectly contributed to the reduction of lipids accumulation in broilers, based on the observations in the present study that a wide range of genes have actively been involved in the biological processes of oxidation of xenobiotic and endogenous compounds, oxo-fatty acid generation and others as discussed in previous sections. Given the fact that the expression of genes coding for enzymes involved in the tricarboxylic acid cycle showed no significant difference between the treatments or specifically within the treatment in response to dietary BG-intervention, the tricarboxylic acid cycle may not be directly affected by dietary BG intervention.

In summary, the present study has shown that dietary BG intervention significantly reduced body fat deposition in young broiler chickens. The decreased fat deposition in BG-fed chickens was in accordance with the level of selected key enzymes involved in lipid metabolism in the serum, abdominal adipose, jejunum and liver. The expression of genes altered by BG treatment, in the jejunum was mainly involved in the biological events for reducing the synthesis, storage, transportation and secretion of lipids, while that in the liver was mostly involved in enhancing the oxidation of ingested lipids and fatty acids. These findings provide a useful reference for the design of new strategies to regulate the ingested energy distribution and to improve animal/human nutrition and health.

## Supporting Information

S1 Table Ingredient composition of experimental diets. (DOCX)

S2 Table Primers for quantitative PCR assays. (DOCX)

S3 Table Profiles of DEGs identified by RNA-Seq analysis in response to butyrate glycerides treatment in the liver of broilers. (DOCX)

S4 Table Profiles of DEGs identified by RNA-Seq analysis in response to butyrate glycerides treatment in the jejunum of broilers. (DOCX)

S5 Table Top networks enriched with differentially expressed genes in response to butyrate glycerides treatment. (DOCX)

S6 Table Top networks enriched with treatment specifically expressed genes in response to butyrate glycerides treatment. (DOCX)

S7 Table Differentially expressed genes involved in lipid metabolism in response to butyrate glycerides treatment in broilers. (DOCX)

S8 Table Treatment specifically expressed genes involved in lipid metabolism in broilers. (DOCX)
