# Correction: Transcriptome Analysis Reveals Regulation of Gene Expression for Lipid Catabolism in Young Broilers by Butyrate Glycerides

**DOI:** 10.1371/journal.pone.0162150

**Published:** 2016-08-25

**Authors:** Fugui Yin, Hai Yu, Dion Lepp, Xuejiang Shi, Xiaojian Yang, Jielun Hu, Steve Leeson, Chengbo Yang, Shaoping Nie, Yongqing Hou, Joshua Gong

The copyright statement appearing with the published article is incorrect. The correct copyright is: 2016 Crown Copyright. This is an open-access article distributed under the terms of the Re-use of Public Sector Information Regulations 2005, which permit unrestricted use, distribution, and reproduction in any medium, provided the original author and source are credited.

The Funding statement in the published article is incorrect. The correct Funding statement should be: Agriculture and Agri-Food Canada and Canadian Poultry Research Council through the Poultry Cluster II Program (Project # J-000264, Dr. Joshua Gong).

There are errors in the Acknowledgements of the published article. The Acknowledgements should read: FY and XY were NSERC Visiting Fellows to the Canadian Federal Government Laboratories. JH was a visiting graduate student financially supported by the China Scholarship Council through the MOE-AAFC Ph.D. Research Program. The funders had no role in study design, data collection and analysis, decision to publish, or preparation of the manuscript.
