# Supplementary material for: Transcriptome Analysis Reveals Regulation of Gene Expression for Lipid Catabolism in Young Broilers by Butyrate Glycerides
Source: PLoS One. 2016 Aug 10;11(8):e0160751. doi: 10.1371/journal.pone.0160751 (PMC4979964; doi:10.1371/journal.pone.0160751)
Supplement: S1 Table — (DOCX) [file pone.0160751.s001.docx]

**Supplemental Table 1. Ingredient composition of experimental diets**

|  | | | | | |
| --- | --- | --- | --- | --- | --- |
| Item | Starter | | | Grower | Finisher |
| Diets | 1 | 2 | 3 | 4 | 5 |
| Ingredient (%) | | | | | |
| Corn | 53.0 | 52.4 | 52.7 | 56.4 | 61.3 |
| Wheat Shorts | 10.0 | 10.0 | 10.0 | 10.0 | 10.0 |
| Soybean meal | 26.2 | 26.2 | 26.2 | 21.9 | 17.4 |
| Meat meal | 6.4 | 6.4 | 6.4 | 5.9 | 5.4 |
| AV Fat | 1.6 | 1.6 | 1.6 | 3.0 | 3.1 |
| DL methionine | 0.3 | 0.3 | 0.3 | 0.3 | 0.3 |
| L-Lysine | 0.2 | 0.2 | 0.2 | 0.2 | 0.2 |
| Salt | 0.3 | 0.3 | 0.3 | 0.3 | 0.3 |
| Limestone | 1.0 | 1.0 | 1.0 | 1.0 | 1.0 |
| Vit-Min PMX | 1.0 | 1.0 | 1.0 | 1.0 | 1.0 |
| Coban | 0.1 | 0.1 | 0.1 | 0.1 | 0.1 |
| Baby C4 ^a^ | 0.0 | 0.3 | 0.0 | 0.0 | 0.0 |
| Mono-butyrin (Mono C4) ^b^ | 0.0 | 0.3 | 0.3 | 0.0 | 0.0 |

^a, b^ Products from SILO (Industria Zootecnica, Florence, Italy). Baby C4 contains 30% mono-butyrin, 50% di-butyrin and 20% tri-butyrin.

The diet schedule for each treatment were:

1) Basal diet-fed group: 0 to 20 d, diet 1; 21 to 33 d, diet 4; 34 to 40 d, diet 5.

2) Butyrate glycerides-fed group: 0 to 7 d, diet 2; 8 to 20 d, diet 3; 21 to 33 d, diet 4; 34 to 40 d, diet 5.
