# Supplementary material for: Transcriptome Analysis Reveals Regulation of Gene Expression for Lipid Catabolism in Young Broilers by Butyrate Glycerides
Source: PLoS One. 2016 Aug 10;11(8):e0160751. doi: 10.1371/journal.pone.0160751 (PMC4979964; doi:10.1371/journal.pone.0160751)
Supplement: S2 Table — (DOCX) [file pone.0160751.s002.docx]

**Supplemental Table 2. Primers for quantitative PCR assays**

|  |  |  |  |  |  |
| --- | --- | --- | --- | --- | --- |
| Gene | GenBank  accession no. | Amplicon size (bp) | Annealing temperature (°C) | Forward primer sequence (5’-3’) | Reverse primer sequence (5’-3’) |
| *CYP8B1* | NM_001005571.1 | 460 | 60 | F: GGGTTACGCACTGGACTTCA | R: GTTCCCTGTCCCTTGGTACG |
| *FABP2* | NM_001007923.1 | 183 | 60 | F: GCAATGGGCGTGAATGTGAT | R: GCCTGAAAGTTCAGTCCCGT |
| *FABP4* | NM_204290.1 | 133 | 61 | F: CTGGCCTGACAAAATGTGCG | R: ATTAGGCTTGGCCACACCAG |
| *LPL* | NM_205282.1 | 303 | 61 | F: GGATCAGCTGGTGAAGTGCT | R: GGCTGGTCTACCTTGGTCAC |
| *MMP-1* | XM_417176.3 | 192 | 60 | F: ATGCTTTTCAGCCAGGGGAA | R: CTTTGGGGTCTGTGTAGGCA |
| *PLIN-1* | NM_001127439.1 | 177 | 60 | F: GACCACAGCAAGGTACACGA | R: GATTGCTGCTGGGAGACCTT |
| *β -actin* | NM_205518.1 | 148 | 61 | F: TGTTACCAACACCCACACCC | R: AGACTGCTGCTGACACCTTC |

# Note: The PCR primers targeting the selected genes were designed with an online program of NCBI primer blast (http://www.ncbi.nlm.nih.gov/tools/primer-blast/) with corresponding mRNA sequences. The PCR products of each gene were verified by sequencing at Lab Services of University of Guelph (Guelph, ON, Canada).

# *β –actin*, actin, beta (HGNC: 132); *CYP8B1*, Cytochrome P450, family 8, subfamily B, polypeptide 1 (HGNC: 2653); *FABP2*, Fatty acid binding protein 2, intestinal (HGNC: 3556); *FABP4*, Fatty acid binding protein 4, adipocyte (HGNC: 3559); *LPL*, Lipoprotein lipase (HGNC: 6677); *MMP1*, Matrix metallopeptidase 1 (HGNC: 7155); *PLIN1*, Perilipin 1 (HGNC: 9076).
