# Supplementary material for: Transcriptome Analysis Reveals Regulation of Gene Expression for Lipid Catabolism in Young Broilers by Butyrate Glycerides
Source: PLoS One. 2016 Aug 10;11(8):e0160751. doi: 10.1371/journal.pone.0160751 (PMC4979964; doi:10.1371/journal.pone.0160751)
Supplement: S3 Table — (DOCX) [file pone.0160751.s003.docx]

**Supplemental Table 3. Profiles of differentially expressed genes in response to butyrate glycerides treatment in the liver of broilers^a^**

|  |  |  |  |  |  |
| --- | --- | --- | --- | --- | --- |
| Gene symbol | Gene name | Average reads (BD-fed group) | Average reads (BG-fed group) | Log2 Fold change | Adjusted *P*-value |
| ACACB | Acetyl-CoA carboxylase beta | 45 | 4 | -3.37 | 7.75E-04 |
| ACTG2 | Actin, gamma 2, smooth muscle, enteric | 270 | 93 | -1.53 | 1.35E-03 |
| AKR1B1L | Aldo-keto reductase family 1, member B1-like | 638 | 1653 | 1.37 | 1.38E-05 |
| ANG | Angiogenin, ribonuclease, RNase A family | 110 | 256 | 1.21 | 1.30E-02 |
| APOA5 | Apolipoprotein A-V | 7873 | 3439 | -1.19 | 7.74E-04 |
| ASPM | Asp (abnormal spindle) homolog, microcephaly associated | 161 | 458 | 1.51 | 6.84E-05 |
| ATP6V0D2 | ATPase, H+ transporting, lysosomal | 8 | 53 | 2.76 | 5.03E-03 |
| ATRNL1 | Attractin-like 1 | 391 | 74 | -2.40 | 9.51E-11 |
| AURKA | Aurora kinase A | 86 | 259 | 1.59 | 6.79E-04 |
| BAIAP2L2 | BAI1-associated protein 2-like 2 | 204 | 76 | -1.42 | 1.38E-02 |
| BDKRB1 | Bradykinin receptor B1 | 25 | 210 | 3.06 | 4.22E-09 |
| BDKRB2 | Bradykinin receptor B2 | 5 | 36 | 2.71 | 1.17E-02 |
| BF2 | Major histocompatibility complex class I antigen | 15004 | 36022 | 1.26 | 1.51E-03 |
| BORA | Bora, aurora kinase A activator | 42 | 117 | 1.49 | 3.53E-02 |
| BRCA1 | Breast cancer 1, early onset | 102 | 276 | 1.43 | 3.10E-03 |
| BST1 | Bone marrow stromal cell antigen 1 | 2202 | 868 | -1.34 | 2.74E-06 |
| BUB1B | BUB1 mitotic checkpoint  serine/threonine kinase B | 81 | 238 | 1.55 | 2.30E-03 |
| C5H14orf37 | Chromosome 5 open reading frame | 350 | 94 | -1.89 | 6.61E-05 |
| CALB1 | Calbindin 1, 28kDa | 160 | 16 | -3.28 | 4.51E-09 |
| CAMP | Cationic antimicrobial protein | 45 | 252 | 2.49 | 1.36E-09 |
| CAPS2 | Calcyphosphine 2 | 18 | 66 | 1.92 | 4.23E-02 |
| CATHL3 | Cathelicidin-3 | 88 | 476 | 2.43 | 7.38E-14 |
| CCNA2 | Cyclin A2 | 233 | 506 | 1.12 | 5.71E-03 |
| CCNB2 | Cyclin B2 | 249 | 639 | 1.36 | 1.26E-04 |
| CCNB3 | Cyclin B3 | 93 | 240 | 1.37 | 5.86E-03 |
| CD55 | CD55 molecule, decay accelerating  factor for complement | 81 | 199 | 1.30 | 3.63E-02 |
| CDC45 | Cell division cycle 45 | 91 | 246 | 1.43 | 4.03E-03 |
| CDCA3 | Cell division cycle associated 3 | 126 | 414 | 1.71 | 4.27E-06 |
| CDHR5 | Cadherin-related family member 5 | 60 | 159 | 1.41 | 3.18E-02 |
| CDK1 | Cyclin-dependent kinase 1 | 241 | 646 | 1.42 | 2.14E-05 |
| CDKN3 | Cyclin-dependent kinase inhibitor 3 | 50 | 135 | 1.43 | 4.10E-02 |
| CENPF | Centromere protein F | 59 | 171 | 1.53 | 1.73E-02 |
| CENPH | Centromere protein H | 93 | 249 | 1.42 | 4.27E-03 |
| CENPI | Centromere protein I | 50 | 140 | 1.48 | 2.96E-02 |
| CENPK | Centromere protein K | 75 | 182 | 1.29 | 2.98E-02 |
| CENPW | Centromere protein W | 43 | 143 | 1.74 | 5.69E-03 |
| CHAC1 | ChaC glutathione-specific gamma-glutamylcyclotransferase 1 | 1119 | 469 | -1.26 | 1.06E-04 |
| CKAP2 | Cytoskeleton associated protein 2 | 157 | 447 | 1.51 | 5.48E-05 |
| CKMT1A | Creatine kinase, mitochondrial 1A | 28 | 3 | -3.41 | 6.44E-03 |
| CKS1B | CDC28 protein kinase regulatory subunit 1B | 164 | 393 | 1.26 | 1.69E-03 |
| COL8A2 | Collagen, type VIII, alpha 2 | 6 | 30 | 2.36 | 3.62E-02 |
| CORO2B | Coronin, actin binding protein | 211 | 26 | -3.04 | 9.14E-10 |
| CP | Ceruloplasmin | 1795 | 5358 | 1.58 | 2.00E-07 |
| CRAT | Carnitine O-acetyltransferase | 4659 | 1992 | -1.23 | 5.98E-05 |
| CRMP1 | Collapsin response mediator protein 1 | 107 | 243 | 1.18 | 3.59E-02 |
| CRYBA2 | Crystallin, beta A2 | 2 | 230 | 6.72 | 5.69E-25 |
| CSF3R | Colony stimulating factor 3 receptor (granulocyte) | 98 | 222 | 1.17 | 3.55E-02 |
| CTBS | Chitobiase, di-N-acetyl- | 525 | 1440 | 1.46 | 2.45E-06 |
| CTSG | Cathepsin G | 279 | 630 | 1.17 | 3.10E-03 |
| CYP1A1 | Cytochrome P450 1A1 | 1150 | 544 | -1.08 | 5.08E-03 |
| CYP2C9 | Cytochrome P-450 2C9 | 635 | 179 | -1.82 | 3.45E-09 |
| CYP8B1 | Cytochrome P450, family 8, subfamily B, polypeptide 1 | 1654 | 530 | -1.64 | 2.87E-09 |
| DHRS13 | Dehydrogenase/reductase (SDR family) member 13 | 644 | 320 | -1.01 | 2.84E-02 |
| DIO2 | Deiodinase, iodothyronine, type II | 3621 | 7517 | 1.05 | 2.40E-02 |
| DLGAP5 | Discs, large (Drosophila) homolog-associated protein 5 | 137 | 353 | 1.37 | 1.63E-03 |
| DPEP1 | Dipeptidase 1 | 26 | 3 | -3.38 | 1.05E-02 |
| DPP4 | Dipeptidyl-peptidase 4 | 694 | 1454 | 1.07 | 2.17E-03 |
| ECT2 | Epithelial cell transforming 2 | 64 | 161 | 1.34 | 4.76E-02 |
| EGR1 | Early growth response 1 | 851 | 2497 | 1.55 | 3.14E-08 |
| EMB | Embigin | 118 | 307 | 1.38 | 3.35E-03 |
| ENPP7 | Ectonucleotide pyrophosphatase/phosphodiesterase 7 | 30 | 4 | -2.91 | 1.64E-02 |
| ERCC6L | Excision repair cross-complementation group 6-like | 58 | 170 | 1.56 | 9.21E-03 |
| FAM72A | Family with sequence similarity 72, member A | 60 | 173 | 1.52 | 7.61E-03 |
| FABP2 | Fatty acid binding protein 2 | 34 | 4 | -2.93 | 9.29E-03 |
| FCGBP | Fc fragment of IgG binding protein | 55 | 6 | -3.27 | 1.81E-04 |
| FOXM1 | Forkhead box M1 | 119 | 254 | 1.10 | 4.81E-02 |
| GAL1 | Gallinacin 1 | 101 | 784 | 2.96 | 2.96E-24 |
| GAL2 | Gallinacin 2 | 93 | 527 | 2.50 | 1.99E-15 |
| GAL4 | Gallinacin-4 | 4 | 33 | 3.02 | 2.60E-03 |
| GAL6 | Gallinacin-6 | 19 | 153 | 3.03 | 6.62E-09 |
| GAL7 | Galactose-1-phosphate | 62 | 318 | 2.36 | 3.27E-10 |
| GAS2 | Growth arrest-specific 2 | 89 | 242 | 1.45 | 3.41E-03 |
| GBP | Guanylate binding protein | 695 | 168 | -2.05 | 3.45E-11 |
| GDAP1 | Ganglioside induced differentiation associated protein 1 | 4 | 36 | 3.04 | 3.53E-03 |
| GIMAP5 | GTPase, IMAP family member 5 | 1115 | 174 | -2.68 | 4.89E-20 |
| GPX3 | Glutathione peroxidase 3 | 40524 | 15814 | -1.36 | 1.45E-03 |
| GRPR | Gastrin-releasing peptide receptor | 3 | 28 | 3.01 | 1.52E-02 |
| GUCY2C | Guanylate cyclase 2C | 363 | 124 | -1.54 | 3.95E-05 |
| HERPUD1 | Homocysteine-inducible, endoplasmic reticulum stress-inducible, ubiquitin-like domain member 1 | 3340 | 1651 | -1.02 | 5.09E-03 |
| HMGB2 | High mobility group box 2 | 167 | 345 | 1.05 | 3.01E-02 |
| HMGCL | 3-hydroxymethyl-3-methylglutaryl-CoA lyase | 5863 | 2773 | -1.08 | 3.35E-03 |
| HMMR | Hyaluronan-mediated motility receptor | 40 | 153 | 1.92 | 1.28E-03 |
| HOGA1 | 4-hydroxy-2-oxoglutarate aldolase 1 | 201 | 72 | -1.47 | 1.08E-02 |
| HS3ST3A1 | Heparan sulfate (glucosamine) 3-O-sulfotransferase 3A1 | 469 | 224 | -1.07 | 2.07E-02 |
| HTATIP2 | HIV-1 Tat interactive protein 2 | 920 | 334 | -1.46 | 7.28E-06 |
| IGJ | Immunoglobulin J polypeptide | 234 | 585 | 1.32 | 3.95E-05 |
| IL22RA2 | Interleukin 22 receptor, alpha 2 | 42 | 7 | -2.54 | 1.17E-02 |
| KCNMB1 | Potassium channel subfamily M regulatory beta subunit 1 | 96 | 352 | 1.88 | 6.63E-06 |
| KIAA1244 | ARFGEF family member 3 | 15 | 65 | 2.11 | 1.43E-02 |
| KIF11 | Kinesin family member 11 | 127 | 359 | 1.50 | 4.01E-04 |
| KIF15 | Kinesin family member 15 | 150 | 341 | 1.19 | 1.15E-02 |
| KIF20A | Kinesin family member 20A | 250 | 530 | 1.09 | 7.00E-03 |
| KIF23 | Kinesin family member 23 | 187 | 435 | 1.22 | 3.74E-03 |
| KIF2C | Kinesin family member 2C | 79 | 214 | 1.44 | 4.28E-03 |
| KIF4A | Kinesin family member 4A | 295 | 788 | 1.42 | 8.28E-06 |
| KNTC1 | Kinetochore associated 1 | 141 | 363 | 1.36 | 2.11E-03 |
| KPNA2 | Karyopherin alpha 2 (RAG cohort 1, importin alpha 1) | 359 | 943 | 1.39 | 6.24E-06 |
| LCN8 | Lipocalin 8 | 72 | 207 | 1.52 | 4.56E-03 |
| LECT2 | Leukocyte cell-derived chemotaxin 2 | 246 | 1597 | 2.70 | 8.95E-26 |
| LOC100857128 | Mucin-2-like | 59 | 11 | -2.44 | 5.30E-03 |
| LOC100857131 | Sperm-associated antigen 4 protein-like | 1136 | 27 | -5.38 | 1.24E-54 |
| LOC100857157 | Uncharacterized | 172 | 9 | -4.26 | 1.37E-12 |
| LOC100857191 | C-C motif chemokine 26-like | 144 | 372 | 1.37 | 2.67E-04 |
| LOC100858007 | Cytochrome P450 2C21-like | 1418 | 417 | -1.77 | 7.58E-11 |
| LOC100858103 | Serum amyloid A protein | 1598 | 6890 | 2.11 | 5.46E-13 |
| LOC100858798 | Nicotinamide riboside kinase 2-like | 48 | 141 | 1.54 | 1.17E-02 |
| LOC100858813 | Myelin-oligodendrocyte glycoprotein-like | 5 | 113 | 4.49 | 2.38E-10 |
| LOC100859069 | Class I histocompatibility antigen, F10 alpha chain-like | 1028 | 289 | -1.83 | 2.87E-09 |
| LOC100859093 | Envelope glycoprotein gp95-like | 27 | 133 | 2.28 | 1.13E-04 |
| LOC100859106 | Envelope glycoprotein gp95-like | 120 | 32 | -1.93 | 1.75E-02 |
| LOC100859177 | Alpha-1-acid glycoprotein 1-like | 1606 | 603 | -1.41 | 1.16E-04 |
| LOC100859282 | Class I histocompatibility antigen, F10 alpha chain-like | 112 | 24 | -2.22 | 4.30E-04 |
| LOC100859304 | Envelope glycoprotein gp95-like | 114 | 32 | -1.84 | 2.94E-02 |
| LOC100859347 | Aryl hydrocarbon receptor-like | 238 | 106 | -1.16 | 4.74E-02 |
| LOC100859413 | Ubiquitin-conjugating enzyme | 89 | 240 | 1.43 | 2.91E-03 |
| LOC100859557 | Condensin-2 complex subunit G2-like | 55 | 156 | 1.49 | 2.04E-02 |
| LOC100859742 | Uncharacterized | 348 | 871 | 1.32 | 5.62E-05 |
| LOC100859797 | Atypical chemokine receptor 4 | 75 | 192 | 1.36 | 3.64E-02 |
| LOC415756 | Uncharacterized | 130 | 453 | 1.80 | 1.72E-06 |
| LOC415922 | Guanylate-binding protein 4-like | 3103 | 1368 | -1.18 | 3.48E-04 |
| LOC416933 | Coiled-coil-helix-coiled-coil-helix domain containing 10 | 5310 | 2598 | -1.03 | 4.89E-03 |
| LOC417083 | Class I histocompatibility antigen, F10 alpha chain-like | 1657 | 158 | -3.39 | 2.61E-33 |
| LOC417253 | Glutamine synthetase-like | 210 | 509 | 1.28 | 3.88E-04 |
| LOC418424 | Uncharacterized | 56 | 255 | 2.18 | 7.09E-07 |
| LOC419677 | Protein FAM49A-like | 446 | 215 | -1.05 | 1.43E-02 |
| LOC421195 | Protocadherin Fat 4-like | 28 | 1 | -5.69 | 1.16E-04 |
| LOC421956 | Zona pellucida glycoprotein-like | 529 | 103 | -2.37 | 2.62E-11 |
| LOC423134 | Proteoglycan 2, bone marrow-like | 29 | 85 | 1.55 | 2.51E-02 |
| LOC423347 | Acyl-CoA synthetase short-chain family member 1-like | 1449 | 686 | -1.08 | 5.52E-04 |
| LOC423943 | Deleted in malignant brain tumors 1-like | 110 | 348 | 1.66 | 1.04E-04 |
| LOC424727 | Structure-specific endonuclease subunit SLX1-like | 310 | 137 | -1.18 | 1.73E-02 |
| LOC425137 | Aldo-keto reductase family 1, member B1-like | 676 | 1544 | 1.19 | 2.75E-04 |
| LOC426385 | Serine/threonine kinase 35-like | 61 | 150 | 1.29 | 4.86E-02 |
| LOC427201 | Rho-guanine nucleotide exchange factor-like | 68 | 193 | 1.50 | 1.17E-02 |
| LOC428505 | SUN domain-containing protein 3-like | 214 | 83 | -1.37 | 3.58E-02 |
| LOC769080 | WD repeat-containing protein 72-like | 3 | 23 | 2.71 | 2.84E-02 |
| LOC769580 | Microsomal triglyceride transfer protein large subunit-like | 2428 | 928 | -1.39 | 2.68E-06 |
| LOC769668 | Estradiol 17-beta-dehydrogenase 2-like | 2822 | 1278 | -1.14 | 9.56E-05 |
| LOC770379 | Small integral membrane protein 3 | 570 | 222 | -1.36 | 3.48E-04 |
| LOC770617 | Transmembrane protein 100-like | 267 | 114 | -1.22 | 3.55E-02 |
| LOC770922 | Deoxyguanosine kinase | 154 | 310 | 1.01 | 3.75E-02 |
| LOC771069 | C-factor-like | 1178 | 434 | -1.44 | 2.30E-06 |
| LOC771349 | Uncharacterized | 115 | 39 | -1.56 | 2.06E-02 |
| LOC771702 | Sperm associated antigen 5 | 157 | 357 | 1.18 | 5.86E-03 |
| LPL | Lipoprotein lipase | 1075 | 512 | -1.07 | 1.63E-03 |
| LYZ | Lysozyme | 23 | 108 | 2.26 | 1.74E-04 |
| MANSC1 | MANSC domain containing 1 | 92 | 262 | 1.52 | 1.88E-03 |
| MAP3K7CL | MAP3K7 C-terminal like | 162 | 492 | 1.60 | 4.75E-06 |
| MEP1A | Meprin A, alpha | 36 | 4 | -3.16 | 4.88E-03 |
| MKI67 | Marker of proliferation Ki-67 | 316 | 771 | 1.29 | 1.59E-04 |
| MMP1 | Matrix metallopeptidase 1 | 109 | 581 | 2.41 | 1.27E-12 |
| MPO | Myeloperoxidase | 29 | 114 | 1.97 | 4.80E-04 |
| MR1 | Major histocompatibility complex class I-related | 1806 | 375 | -2.27 | 2.00E-16 |
| MSLN | Mesothelin | 92 | 28 | -1.71 | 2.94E-02 |
| MUC2 | Mucin 2 | 53 | 6 | -3.24 | 2.48E-04 |
| NCAPG | Non-SMC condensin I complex, subunit G | 93 | 247 | 1.41 | 5.09E-03 |
| NCAPH | Non-SMC condensin I complex, subunit H | 110 | 251 | 1.19 | 2.51E-02 |
| NDC80 | Kinetochore protein NDC80 | 68 | 201 | 1.56 | 4.55E-03 |
| NEBL | Nebulette | 111 | 242 | 1.12 | 2.45E-02 |
| NEK10 | NIMA-related kinase 10 | 2 | 30 | 4.24 | 8.91E-04 |
| NEK2 | NIMA-related kinase 2 | 33 | 104 | 1.67 | 2.26E-02 |
| NME4 | NME/NM23 nucleoside diphosphate kinase 4 | 21341 | 9882 | -1.11 | 5.86E-03 |
| NUSAP1 | Nucleolar and spindle associated protein 1 | 105 | 258 | 1.30 | 1.20E-02 |
| OLFM4 | Olfactomedin 4 | 13 | 1 | -4.58 | 2.77E-02 |
| OTOP1 | Otopetrin 1 | 9 | 67 | 2.90 | 5.22E-04 |
| OVCH2 | Ovochymase 2 | 215 | 433 | 1.01 | 2.14E-02 |
| PACSIN1 | Protein kinase C and casein kinase substrate in neurons 1 | 148 | 42 | -1.83 | 6.85E-03 |
| PAFAH2 | Platelet-activating factor acetylhydrolase 2 | 1187 | 545 | -1.12 | 3.87E-04 |
| PBK | PDZ binding kinase | 134 | 406 | 1.60 | 2.38E-05 |
| PCSK9 | Proprotein convertase subtilisin/kexin type 9 | 307 | 86 | -1.83 | 2.06E-05 |
| PDE11A | Phosphodiesterase 11A | 26 | 5 | -2.49 | 3.45E-02 |
| PGCP | Carboxypeptidase Q | 205 | 462 | 1.17 | 2.03E-03 |
| PLACL2 | Placenta-specific gene 8-like 2 | 572 | 1251 | 1.13 | 1.06E-05 |
| PLCD4 | Phospholipase C, delta 4 | 1099 | 211 | -2.38 | 5.80E-18 |
| PLK1 | Polo-like kinase 1 | 122 | 394 | 1.70 | 7.06E-06 |
| PPP3CA | Protein phosphatase 3 (formerly 2B), catalytic subunit, alpha isoform | 257 | 546 | 1.09 | 2.35E-02 |
| PRC1 | Protein regulator of cytokinesis 1 | 193 | 416 | 1.11 | 6.88E-03 |
| PRR13 | Proline rich 13 | 1958 | 614 | -1.67 | 6.18E-08 |
| PTTG1 | Pituitary tumor-transforming 1 | 40 | 127 | 1.68 | 1.27E-02 |
| RAB23 | Member RAS oncogene family 23 | 28 | 125 | 2.18 | 4.81E-04 |
| RBP2 | Retinol binding protein 2, cellular | 24 | 2 | -3.54 | 9.82E-03 |
| RIMBP2 | RIMS binding protein 2 | 120 | 279 | 1.21 | 3.37E-02 |
| RSAD2 | Radical S-adenosyl methionine domain containing 2 | 102 | 230 | 1.17 | 2.21E-02 |
| S100A9 | S100 calcium binding protein A9 | 81 | 536 | 2.72 | 4.11E-17 |
| SELP | Selectin P | 95 | 215 | 1.19 | 3.55E-02 |
| SERPINB10 | Serpin peptidase inhibitor, clade B | 24 | 106 | 2.12 | 7.35E-04 |
| SI | Sucrase-isomaltase | 110 | 9 | -3.57 | 5.35E-08 |
| SLC16A5 | Solute carrier family 16 (monocarboxylate transporter), member 5 | 772 | 342 | -1.18 | 1.38E-03 |
| SLC19A1 | Solute carrier family 19 (folate transporter), member 1 | 5248 | 2516 | -1.06 | 3.04E-03 |
| SLC23A1 | Solute carrier family 23 (ascorbic acid transporter), member 1 | 462 | 223 | -1.05 | 1.08E-02 |
| SLC25A47 | Solute carrier family 25, member 47 | 1013 | 492 | -1.04 | 3.25E-03 |
| SMC2 | Structural maintenance of chromosomes 2 | 466 | 1250 | 1.42 | 2.56E-06 |
| SMC4 | Structural maintenance of chromosomes 4 | 412 | 855 | 1.05 | 3.90E-03 |
| SMYD1 | SET and MYND domain containing 1 | 99 | 283 | 1.52 | 2.06E-04 |
| SPAG4 | Sperm associated antigen 4 | 2151 | 83 | -4.69 | 3.53E-54 |
| SPINK2 | Serine peptidase inhibitor, Kazal type 2 (acrosin-trypsin inhibitor) | 20 | 101 | 2.35 | 9.56E-05 |
| SPIRE2 | Spire-type actin nucleation factor 2 | 1735 | 825 | -1.07 | 1.82E-03 |
| SRGAP3 | SLIT-ROBO Rho GTPase activating protein 3 | 74 | 183 | 1.30 | 2.94E-02 |
| SSTR1 | Somatostatin receptor 1 | 79 | 373 | 2.23 | 4.84E-09 |
| STMN1 | Stathmin 1 | 306 | 620 | 1.02 | 5.03E-03 |
| SUSD3 | Sushi domain containing 3 | 250 | 58 | -2.11 | 4.41E-06 |
| TECTA | Tectorin alpha | 191 | 66 | -1.54 | 5.86E-03 |
| TGM3 | Transglutaminase 3 | 13 | 106 | 3.06 | 6.30E-07 |
| THY1 | Thy-1 cell surface antigen | 141 | 42 | -1.76 | 2.89E-03 |
| TK1 | Thymidine kinase 1, soluble | 221 | 458 | 1.05 | 6.28E-03 |
| TLR6 | Toll-like receptor 6 | 97 | 229 | 1.24 | 3.62E-02 |
| TNIK | TRAF2 and NCK interacting kinase | 343 | 785 | 1.19 | 2.31E-03 |
| TOP2A | Topoisomerase (DNA) II alpha 170kDa | 617 | 1519 | 1.30 | 7.93E-06 |
| TPX2 | Microtubule-associated | 185 | 513 | 1.47 | 3.95E-05 |
| UNC13D | Unc-13 homolog D | 147 | 408 | 1.47 | 2.52E-04 |

**^a^** Determined by TopHat (v2.0.8) analysis; n = 2, each sample was a combined sample from three chickens.
