# Supplementary material for: Transcriptome Analysis Reveals Regulation of Gene Expression for Lipid Catabolism in Young Broilers by Butyrate Glycerides
Source: PLoS One. 2016 Aug 10;11(8):e0160751. doi: 10.1371/journal.pone.0160751 (PMC4979964; doi:10.1371/journal.pone.0160751)
Supplement: S4 Table — (DOCX) [file pone.0160751.s004.docx]

**Supplemental Table 4. Profiles of differentially expressed genes in response to butyrate glycerides treatment in the jejunum of broilers^a^**

|  |  |  |  |  |  |
| --- | --- | --- | --- | --- | --- |
| Gene symbol | Gene name | Average reads (BD-fed group) | Average reads (BG-fed group) | Log_2_ fold- change | Adjusted *P*-value |
| A2M | Alpha-2-macroglobulin | 31 | 226 | 2.85 | 2.12E-13 |
| ACTN2 | Actinin, alpha 2 | 6 | 29 | 2.40 | 4.58E-02 |
| ADH1C | Alcohol dehydrogenase 1C (class I), gamma polypeptide | 373 | 1942 | 2.38 | 5.98E-30 |
| ANK1 | Ankyrin 1, erythrocytic | 103 | 35 | -1.54 | 1.96E-02 |
| ASPA | Aspartoacylase | 507 | 223 | -1.18 | 7.20E-05 |
| BF1 | MHC BF1 class I | 61156 | 30479 | -1.00 | 1.33E-02 |
| CA6 | Carbonic anhydrase VI | 350 | 155 | -1.18 | 1.24E-03 |
| CA7 | Carbonic anhydrase VII | 588 | 1263 | 1.10 | 6.07E-06 |
| CASR | Calcium-sensing receptor | 37 | 129 | 1.81 | 6.72E-04 |
| CLCN1 | Chloride channel, voltage-sensitive 1 | 187 | 57 | -1.72 | 9.43E-05 |
| CMPK2 | Cytidine monophosphate (UMP-CMP) kinase 2, mitochondrial | 4906 | 2209 | -1.15 | 6.64E-08 |
| CTSG | Cathepsin G | 478 | 1038 | 1.12 | 6.87E-06 |
| CYP2C21L | Cytochrome P450 2C21-like | 213 | 974 | 2.19 | 6.94E-21 |
| CYP2C9 | Cytochrome P-450 2C9 | 71 | 312 | 2.14 | 2.03E-10 |
| ENS-1 | Embryonic Normal Stem cell-1 | 27 | 130 | 2.30 | 5.18E-06 |
| FABP4 | Fatty acid binding protein 4, adipocyte | 599 | 204 | -1.55 | 3.52E-08 |
| G0S2 | G0/G1 switch 2 | 176 | 66 | -1.42 | 4.05E-03 |
| GAL3ST2 | Galactose-3-O-sulfotransferase 2 | 24 | 3 | -2.88 | 1.76E-02 |
| HBAA | Hemoglobin, alpha 1 | 5296 | 626 | -3.08 | 3.91E-52 |
| HBE1 | Hemoglobin, epsilon 1 | 26 | 3 | -2.94 | 1.01E-02 |
| HBG2 | Hemoglobin, gamma G | 5570 | 689 | -3.02 | 1.68E-50 |
| HBM | Hemoglobin | 1809 | 192 | -3.24 | 2.50E-49 |
| HSD3B7 | Hydroxy-delta-5-steroid dehydrogenase,  3 beta- and steroid delta-isomerase 7 | 469 | 85 | -2.47 | 1.47E-17 |
| IFIT5 | Interferon-induced protein with tetratricopeptide  repeats 5 | 2890 | 951 | -1.60 | 3.01E-15 |
| KIFC1 | Kinesin family member C1 | 588 | 230 | -1.35 | 1.90E-06 |
| LOC100857280 | Alcohol dehydrogenase 1-like | 382 | 1614 | 2.08 | 2.26E-22 |
| LOC100858232 | E3 ubiquitin-protein ligase HERC4-like | 2559 | 1034 | -1.31 | 5.81E-10 |
| LOC100858355 | Histone H5-like | 143 | 35 | -2.02 | 3.55E-05 |
| LOC100858381 | Chromosome 11 open reading frame, human C19orf12-like | 475 | 232 | -1.03 | 2.34E-03 |
| LOC100858529 | Myelin-oligodendrocyte glycoprotein-like | 121 | 40 | -1.62 | 4.14E-03 |
| LOC100858780 | C-type lectin-like receptor 3 | 29 | 4 | -2.88 | 7.89E-03 |
| LOC100858813 | Myelin-oligodendrocyte glycoprotein-like | 55 | 788 | 3.83 | 1.05E-43 |
| LOC100859069 | Class I histocompatibility antigen, F10 alpha chain-like | 2729 | 318 | -3.10 | 2.50E-49 |
| LOC100859272 | AP four-disulfide core domain protein 2-like | 456 | 203 | -1.17 | 1.29E-04 |
| LOC100859282 | Class I histocompatibility antigen, F10 alpha chain-like | 350 | 115 | -1.61 | 1.76E-06 |
| LOC100859786 | Uncharacterized | 15 | 187 | 3.66 | 7.94E-16 |
| LOC100859855 | Uncharacterized | 1088 | 387 | -1.49 | 1.50E-10 |
| LOC415325 | Uncharacterized | 127 | 38 | -1.73 | 1.28E-03 |
| LOC415756 | Uncharacterized | 497 | 230 | -1.11 | 5.25E-04 |
| LOC417083 | Class I histocompatibility antigen, F10 alpha chain-like | 4530 | 329 | -3.78 | 1.59E-72 |
| LOC417973 | Uncharacterized | 1213 | 3125 | 1.37 | 1.54E-11 |
| LOC418892 | Complement C4-like | 137 | 51 | -1.43 | 1.89E-02 |
| LOC420108 | Uncharacterized | 1185 | 452 | -1.39 | 1.59E-09 |
| LOC422509 | Viral interleukin-8 homolog | 27 | 4 | -2.75 | 1.55E-02 |
| LOC425214 | Flagellar attachment zone protein 1-like | 344 | 45 | -2.93 | 2.02E-18 |
| LOC425531 | Ras-related protein Rab-18-B-like | 259 | 122 | -1.08 | 2.26E-02 |
| LOC426155 | Uncharacterized | 41 | 8 | -2.30 | 1.76E-02 |
| LOC428505 | SUN domain-containing protein 3-like | 29 | 1 | -4.86 | 1.68E-05 |
| LOC431047 | Nuclear receptor subfamily 1,  group D, member 1 | 48 | 141 | 1.56 | 3.21E-03 |
| LOC768349 | Myelin-oligodendrocyte glycoprotein-like | 167 | 26 | -2.66 | 3.66E-09 |
| LOC768351 | MHC class II antigen | 186 | 75 | -1.30 | 9.58E-03 |
| LOC770082 | Heat shock 70kD protein 12B | 1137 | 2798 | 1.30 | 2.03E-10 |
| LOC770450 | Serine protease inhibitor Kazal-type 6-like | 365 | 123 | -1.57 | 1.17E-06 |
| LOC770611 | Uncharacterized | 1 | 57 | 5.85 | 3.18E-10 |
| LOC770612 | Interferon-induced transmembrane protein 1-like | 15619 | 6397 | -1.29 | 4.49E-08 |
| LPL | Lipoprotein lipase | 414 | 201 | -1.04 | 7.47E-03 |
| LY6E | Lymphocyte antigen 6 complex, locus E | 18038 | 5494 | -1.72 | 1.25E-14 |
| LYG2 | Lysozyme G-like 2 | 437 | 63 | -2.80 | 5.59E-20 |
| MMP1 | Matrix metallopeptidase 1 (interstitial collagenase) | 54 | 195 | 1.85 | 6.87E-06 |
| MR1 | Major Histocompatibility Complex, Class I-Related | 4929 | 1014 | -2.28 | 5.98E-30 |
| MYH6 | Myosin, heavy chain 6 | 19 | 172 | 3.21 | 4.06E-12 |
| MYH8 | Myosin, heavy chain 8 | 9 | 87 | 3.25 | 2.25E-07 |
| MYL1 | Myosin, light chain 1 | 17 | 86 | 2.32 | 2.66E-04 |
| NECAB1 | N-terminal EF-hand calcium binding protein 1 | 262 | 127 | -1.04 | 2.36E-02 |
| NR1D2 | Nuclear receptor subfamily 1,  group D, member 2 | 1139 | 541 | -1.07 | 1.74E-05 |
| OASL | 2'-5'-oligoadenylate synthetase-like | 20245 | 7886 | -1.36 | 8.14E-09 |
| PATL2 | Protein associated with topoisomerase II homolog 2 | 266 | 113 | -1.24 | 2.14E-03 |
| PLACL2 | Placenta-specific gene 8-like 2 | 5708 | 2150 | -1.41 | 1.69E-11 |
| PLIN1 | Perilipin 1 | 601 | 201 | -1.58 | 1.40E-08 |
| RAG2 | Recombination activating gene 2 | 185 | 5 | -5.15 | 5.07E-23 |
| REG4 | Regenerating islet-derived family, member 4 | 2420 | 5320 | 1.14 | 1.16E-07 |
| RSAD2 | Radical S-adenosyl methionine domain containing 2 | 689 | 262 | -1.40 | 1.43E-07 |
| SH3TC2 | SH3 domain and tetratricopeptide repeats 2 | 104 | 244 | 1.24 | 2.76E-03 |
| SLBP | Stem-loop binding protein | 429 | 203 | -1.08 | 1.89E-03 |
| SLC16A4 | Solute carrier family 16, member 4 | 124 | 310 | 1.32 | 6.42E-04 |
| SLC2A5 | Solute carrier family 2 (facilitated glucose/fructose transporter), member 5 | 4327 | 2054 | -1.07 | 9.62E-07 |
| SLC5A12 | Solute carrier family 5 (sodium/monocarboxylate cotransporter), member 12 | 805 | 260 | -1.63 | 1.92E-11 |
| SLC5A8 | Solute carrier family 5 (sodium/monocarboxylate cotransporter), member 8 | 46 | 437 | 3.24 | 1.39E-24 |
| SLITRK3 | SLIT and NTRK-like family, member 3 | 220 | 93 | -1.24 | 8.63E-03 |
| THRSP | Thyroid hormone responsive | 199 | 64 | -1.63 | 2.66E-04 |
| TMPRSS15 | Transmembrane protease, serine 15 | 30 | 156 | 2.36 | 3.91E-07 |
| TNNI2 | Troponin I type 2 (skeletal, fast) | 7 | 54 | 2.90 | 2.11E-04 |
| TNNT3 | Troponin T type 3 (skeletal, fast) | 26 | 91 | 1.80 | 9.00E-03 |
| TTC38 | Tetratricopeptide repeat domain 38 | 446 | 1149 | 1.37 | 3.13E-09 |
| USP18 | Ubiquitin specific peptidase 18 | 5071 | 1300 | -1.96 | 5.07E-23 |
| WIF1 | WNT inhibitory factor 1 | 135 | 52 | -1.38 | 1.80E-02 |
| ZNF692 | Zinc finger protein 692 | 267 | 109 | -1.29 | 1.29E-03 |

^a^ Determined by TopHat (v2.0.8) analysis; n = 2, each sample was a combined sample from three chickens.
