# Supplementary material for: Transcriptome Analysis Reveals Regulation of Gene Expression for Lipid Catabolism in Young Broilers by Butyrate Glycerides
Source: PLoS One. 2016 Aug 10;11(8):e0160751. doi: 10.1371/journal.pone.0160751 (PMC4979964; doi:10.1371/journal.pone.0160751)
Supplement: S5 Table — (DOCX) [file pone.0160751.s005.docx]

**Supplemental Table 5. Top networks enriched with differentially expressed genes in response to butyrate glycerides treatment ^a^**

|  |  |  |  |
| --- | --- | --- | --- |
| Tissue | ID | Associated network functions | Score |
| Jejunum | 1 | Lipid metabolism, small molecule biochemistry, immunological disease | 84 |
|  | 2 | Cellular compromise, immunological disease, cell death and survival | 59 |
|  | 3 | Cancer, embryonic development, neurological disease | 2 |
|  | 4 | Cell cycle, reproductive system development and function, tissue development | 2 |
|  | 5 | Cancer, DNA replication, recombination, and repair, Developmental Disorder | 2 |
| Liver | 1 | Inflammatory Disease, Respiratory Disease, Hematological System Development and Function | 78 |
|  | 2 | Carbohydrate Metabolism, Lipid Metabolism, Molecular Transport | 5 |
|  | 3 | Cellular Development, Cellular Growth and Proliferation, Hematological System Development and Function | 2 |
|  | 4 | Cell Cycle, Tissue Development, Cellular Assembly and Organization | 2 |

^a^ Determined by IPA analysis; n = 2, each sample was a combined sample from three chickens.
