# Supplementary material for: Transcriptome Analysis Reveals Regulation of Gene Expression for Lipid Catabolism in Young Broilers by Butyrate Glycerides
Source: PLoS One. 2016 Aug 10;11(8):e0160751. doi: 10.1371/journal.pone.0160751 (PMC4979964; doi:10.1371/journal.pone.0160751)
Supplement: S6 Table — (DOCX) [file pone.0160751.s006.docx]

**Supplemental Table 6. Top networks enriched with treatment specifically expressed genes in response to butyrate glycerides treatment ^a^**

|  |  |  |  |  |
| --- | --- | --- | --- | --- |
| Treatment | Tissue | ID | Associated network functions | Score |
| BG-fed group | Jejunum | 1 | Cell-to-cell signaling and interaction, nucleic acid metabolism, small molecule biochemistry | 53 |
|  |  | 2 | Connective tissue disorders, developmental disorder, skeletal and muscular disorders | 51 |
|  |  | 3 | Free radical scavenging, small molecule biochemistry, hereditary disorder | 46 |
|  |  | 4 | Cellular function and maintenance, gene expression, cell death and survival | 8 |
|  |  | 5 | Cell cycle, reproductive system development and function, cellular development | 2 |
|  | Liver | 1 | Developmental disorder, ophthalmic disease, cellular compromise | 89 |
|  |  | 2 | Auditory disease, molecular transport, cardiovascular disease | 64 |
|  |  | 3 | Cell-to-cell signaling and interaction, nutritional disease, psychological disorders | 61 |
|  |  | 4 | Cellular assembly and organization, Cell-to-cell signaling and interaction, skeletal and muscular system development and function | 59 |
|  |  | 5 | Inflammatory response, cell-to-cell signaling and interaction, Hematological system development and function | 30 |
| BD-fed group | Jejunum | 1 | Skeletal and muscular disorders, behavior, connective tissue disorders | 75 |
|  |  | 2 | Cellular compromise, inflammatory response, hematological disease | 44 |
|  |  | 3 | Cell morphology, cellular assembly and organization, cellular development | 41 |
|  |  | 4 | Cell signaling, nucleic acid metabolism, small molecule biochemistry | 26 |
|  |  | 5 | Cell-to-cell signaling and Interaction, cellular development, embryonic development | 2 |
|  | Liver | 1 | Behavior, neurological disease, dermatological diseases and conditions | 58 |
|  |  | 2 | Lipid metabolism, small molecule biochemistry, vitamin and mineral metabolism | 54 |
|  |  | 3 | Cell cycle, carbohydrate metabolism, energy production | 51 |
|  |  | 4 | Lipid metabolism, molecular transport, small molecule biochemistry | 10 |
|  |  | 5 | Auditory disease, cell morphology, cell-to-cell signaling and interaction | 2 |

^a^ Determined by IPA analysis; n = 2, each sample was a combined sample from three chickens.

BD, Basal diet; BG, Butyrate glycerides.
