# Supplementary material for: Transcriptome Analysis Reveals Regulation of Gene Expression for Lipid Catabolism in Young Broilers by Butyrate Glycerides
Source: PLoS One. 2016 Aug 10;11(8):e0160751. doi: 10.1371/journal.pone.0160751 (PMC4979964; doi:10.1371/journal.pone.0160751)
Supplement: S7 Table — (DOCX) [file pone.0160751.s007.docx]

**Supplemental Table 7. Differentially expressed genes involved in lipid metabolism in response to butyrate glycerides treatment in broilers ^a^**

|  |  |  |  |  |  |
| --- | --- | --- | --- | --- | --- |
| Tissue | Function Annotation | Up-regulated gene | Down-regulated gene | No. of genes | *P*-Value |
| Jejunum | Abnormal quantity of cholesterol | *---* | *LPL* | 1 | 4.52E-02 |
|  | Abnormal quantity of fatty acid | *---* | *PLIN1* | 1 | 4.84E-02 |
|  | Abnormal quantity of lipid | *---* | *LPL, PLIN1* | 2 | 3.43E-02 |
|  | Accumulation of acylglycerol | *CASR* | *FABP4, LPL, PLIN1* | 4 | 1.91E-05 |
|  | Accumulation of ceramide | *CASR* | *---* | 1 | 4.21E-02 |
|  | Accumulation of cholesterol ester | *---* | *FABP4* | 1 | 3.57E-02 |
|  | Accumulation of diacylglycerol | *CASR* | *---* | 1 | 6.58E-03 |
|  | Accumulation of phosphatidylinositol phosphate | *CASR* | *---* | 1 | 9.86E-03 |
|  | Accumulation of retinyl ester | *---* | *LPL* | 1 | 1.31E-02 |
|  | Accumulation of triacylglycerol | *---* | *FABP4, LPL, PLIN1* | 3 | 4.67E-04 |
|  | Activation of fatty acid | *CYP2C9* | *---* | 1 | 2.93E-02 |
|  | Beta-oxidation of palmitic acid | *---* | *PLIN1* | 1 | 3.25E-02 |
|  | Breakdown of fatty acid | *---* | *LPL* | 1 | 6.58E-03 |
|  | Catabolism of lipid | *---* | *LPL, PLIN1* | 2 | 8.29E-03 |
|  | Catabolism of triacylglycerol | *---* | *LPL* | 1 | 1.64E-02 |
|  | Clearance of triacylglycerol | *---* | *LPL* | 1 | 1.96E-02 |
|  | Concentration of cholesterol | *---* | *FABP4, LPL, RAG2* | 3 | 1.92E-02 |
|  | Concentration of fatty acid | *---* | *FABP4, LPL, PLIN1* | 3 | 1.80E-02 |
|  | Concentration of stearic acid | *---* | *FABP4* | 1 | 1.64E-02 |
|  | Conversion of lipid | *CYP2C9* | *FABP4* | 2 | 3.36E-02 |
|  | Conversion of retinaldehyde | *CYP2C9* | *---* | 1 | 9.86E-03 |
|  | Conversion of vitamin A | *CYP2C9* | *---* | 1 | 9.86E-03 |
|  | Fatty acid metabolism | *CASR, CYP2C9* | *FABP4, LPL* | 4 | 3.38E-02 |
|  | Homeostasis of cholesterol | *CASR* | *FABP4* | 2 | 7.18E-03 |
|  | Homeostasis of lipid | *CASR* | *FABP4, LPL, PLIN1* | 4 | 2.91E-04 |
|  | Homeostasis of triacylglycerol | *--* | *LPL* | 1 | 1.64E-02 |
|  | Hydrolysis of 1,2-dipalmitoylphosphatidylcholine | *--* | *LPL* | 1 | 6.58E-03 |
|  | Hydrolysis of fatty acid | *---* | *FABP4, LPL, PLIN1* | 3 | 1.40E-05 |
|  | Hydrolysis of phosphatidylethanolamine | *---* | *LPL* | 1 | 9.86E-03 |
|  | Hydrolysis of retinyl ester |  | *LPL* | 1 | 1.96E-02 |
|  | Hydrolysis of triacylglycerol | *---* | *LPL, PLIN1* | 2 | 1.38E-03 |
|  | Hydrolysis of triolein | *---* | *LPL* | 1 | 9.86E-03 |
|  | Hydroxylation of lauric acid | *CYP2C9* | *---* | 1 | 6.58E-03 |
|  | Lipolysis of adipocytes | *---* | *PLIN1* | 1 | 2.61E-02 |
|  | Lipolysis of fat | *---* | *PLIN1* | 1 | 3.30E-03 |
|  | Lipolysis of fatty acid | *---* | *FABP4, PLIN1* | 2 | 1.04E-04 |
|  | Lipolysis of fibroblast cell lines | *---* | *PLIN1* | 1 | 3.57E-02 |
|  | Lipolysis of triacylglycerol | *---* | *PLIN1* | 1 | 1.31E-02 |
|  | Metabolism of 11,12-epoxyeicosatrienoic acid | *CYP2C9* | *---* | 1 | 9.86E-03 |
|  | Metabolism of 12-hydroxyeicosatetraenoic acid | *CYP2C9* | *---* | 1 | 9.86E-03 |
|  | Metabolism of 14,15-epoxyeicosatrienoic acid | *CYP2C9* | *---* | 1 | 1.31E-02 |
|  | Metabolism of 17-alpha-ethinylestradiol | *CYP2C9* | *---* | 1 | 9.86E-03 |
|  | Metabolism of 20-hydroxyeicosatetraenoic acid | *CYP2C9* | *---* | 1 | 6.58E-03 |
|  | Metabolism of 8,9-epoxyeicosatrienoic acid | *CYP2C9* | *---* | 1 | 9.86E-03 |
|  | Metabolism of arachidonic acid | *CYP2C9* | *---* | 1 | 3.57E-02 |
|  | Metabolism of linoleic acid | *CYP2C9* | *---* | 1 | 1.64E-02 |
|  | Metabolism of tretinoin | *ADH1C, CYP2C9* | *--* | 2 | 4.52E-02 |
|  | Oxidation of 2-hydroxyestradiol | *CYP2C9* | *---* | 1 | 9.86E-03 |
|  | Oxidation of beta-estradiol | *CYP2C9* | *---* | 1 | 1.96E-02 |
|  | Oxidation of fatty acid | *ADH1C, CYP2C9* | *LPL, PLIN1* | 4 | 4.89E-03 |
|  | Oxidation of fluvastatin | *CYP2C9* | *---* | 1 | 3.30E-03 |
|  | Oxidation of triacylglycerol | *---* | *LPL* | 1 | 6.58E-03 |
|  | Quantity of lysophosphatidylcholine | *---* | *LPL* | 1 | 2.93E-02 |
|  | Quantity of non-esterified fatty acid | *---* | *FABP4* | 1 | 2.29E-02 |
|  | Quantity of palmitoleic acid | *---* | *FABP4* | 1 | 9.86E-03 |
|  | Release of fatty acid | *CASR* | *FABP4* | 2 | 4.32E-02 |
|  | Secretion of prostaglandin E2 | *---* | *FABP4* | 1 | 3.89E-02 |
|  | Secretion of testosterone | *CTSG* | *---* | 1 | 3.89E-02 |
|  | Storage of cholesterol | *---* | *LPL* | 1 | 3.89E-02 |
|  | Storage of fatty acid | *---* | *FABP4* | 1 | 6.58E-03 |
|  | Storage of lipid | *---* | *FABP4, LPL, PLIN1* | 3 | 3.11E-04 |
|  | Storage of triacylglycerol | *---* | *PLIN1* | 1 | 1.64E-02 |
|  | Synthesis of acylglycerol | *---* | *LPL, PLIN1* | 3 | 1.80E-02 |
|  | Synthesis of phosphatidylinositol phosphate | *CASR* | *---* | 1 | 3.57E-02 |
|  | Synthesis of triacylglycerol | *---* | *LPL, PLIN1* | 2 | 6.14E-03 |
|  | Transmission of retinoid | *---* | *LPL* | 1 | 3.30E-03 |
|  | Transport of lipid | *---* | *FABP4, LPL* | 2 | 4.40E-02 |
|  | Transport of palmitic acid | *---* | *FABP4* | 1 | 3.89E-02 |
|  | Transport of triacylglycerol | *---* | *LPL* | 1 | 1.31E-02 |
|  | Uptake of cholesterol ester | *A2M* | *LPL* | 2 | 4.62E-04 |
|  | Uptake of lipid | *A2M* | *FABP4, LPL* | 3 | 3.70E-03 |
|  | Uptake of retinoid | *---* | *LPL* | 1 | 1.64E-02 |
|  | Uptake of triacylglycerol | *---* | *LPL* | 1 | 2.61E-02 |
|  | Utilization of fatty acid | *---* | *LPL* | 1 | 1.31E-02 |
| Liver | Abnormal quantity of lipid | EGR1 | CYP8B1, FABP2, LPL, RBP2 | 5 | 5.50E-03 |
|  | Abnormal quantity of progesterone | EGR1 | --- | 1 | 1.28E-02 |
|  | Conversion of retinaldehyde | --- | CYP1A1, CYP2C9 | 2 | 4.82E-04 |
|  | Conversion of vitamin A | --- | CYP1A1, CYP2C9 | 2 | 4.82E-04 |
|  | Fatty acid metabolism | AKR1B1L, BRCA1, EGR1, MPO | ACACB, APOA5, CALB1, CRAT, CYP1A1, CYP2C9, FABP2, HMGCL, LPL | 13 | 8.77E-03 |
|  | Localization of oleic acid | --- | CRAT | 1 | 1.28E-02 |
|  | Metabolism of 17-alpha-ethinylestradiol | --- | CYP1A1, CYP2C9 | 2 | 4.82E-04 |
|  | Metabolism of 20-hydroxyeicosatetraenoic acid | --- | CYP1A1, CYP2C9 | 2 | 1.62E-04 |
|  | Metabolism of arachidonic acid | --- | CYP1A1, CYP2C9 | 2 | 8.27E-03 |
|  | Metabolism of retinoid | --- | CYP1A1, CYP2C9, RBP2 | 3 | 7.04E-03 |
|  | Metabolism of tretinoin | --- | CYP1A1, CYP2C9 | 2 | 1.33E-02 |
|  | Metabolism of vitamin A | --- | CYP1A1, RBP2 | 2 | 8.27E-03 |
|  | Oxidation of 2-hydroxyestradiol | --- | CYP1A1, CYP2C9 | 2 | 4.82E-04 |
|  | Oxidation of beta-estradiol | --- | CYP1A1, CYP2C9 | 2 | 2.35E-03 |
|  | Oxidation of fluvastatin | --- | CYP2C9 | 1 | 1.28E-02 |
|  | Production of 2-hydroxyestradiol | --- | CYP1A1 | 1 | 1.28E-02 |
|  | Transmission of retinoid | --- | LPL | 1 | 1.28E-02 |
|  | Utilization of fatty acid | --- | FABP2, LPL | 2 | 9.56E-04 |

**^a^** Determined by IPA analysis; n = 2, each sample was a combined sample from three chickens. The gene expression in the BG-fed chickens was compared to that of BD-fed birds (BG/BD).

Note: *A2M*, Alpha-2-macroglobulin (HGNC: 7); *ACACB*, Acetyl-CoA carboxylase beta (HGNC: 85); *ADH1C*, Alcohol dehydrogenase 1C (class I), gamma polypeptide (HGNC: 251); *AKR1B1*, Aldo-keto reductase family 1, member B1 (aldose reductase) (HGNC: 381); *APOA5*, Apolipoprotein A-V (HGNC: 17288); *BRCA1*, Breast cancer 1, early onset (HGNC: 1100); *CALB1*, Calbindin 1, 28kDa (HGNC: 1434); *CASR*, Calcium-sensing receptor (HGNC: 1514); *CRAT*, Carnitine O-acetyltransferase (HGNC: 2342); *CTSG*, Cathepsin G (HGNC: 2532); *CYP1A1*, Cytochrome P450, family 1, subfamily A, polypeptide 1 (HGNC: 2595); *CYP2C9*, Cytochrome P450, family 2, subfamily C, polypeptide 9 (HGNC: 2623); *CYP8B1*, Cytochrome P450, family 8, subfamily B, polypeptide 1 (HGNC: 2653); *EGR1*, Early growth response 1 (HGNC: 3238); *FABP2*, Fatty acid binding protein 2 intestinal (HGNC: 3556); *FABP4*, Fatty acid binding protein 4, adipocyte (HGNC: 3559); *HMGCL*, 3-hydroxymethyl-3-methylglutaryl-CoA lyase (HGNC: 5005); *LPL*, lipoprotein lipase (HGNC: 6677); *MOG*, myelin oligodendrocyte glycoprotein (HGNC: 7197); *MPO*, Myeloperoxidase (HGNC: 7218); *PLIN1*, Perilipin 1 (HGNC: 9076); *RAG2*, Recombination activating gene 2 (HGNC: 9832); *RBP2*, Retinol binding protein 2, cellular (HGNC: 9920).
