# Supplementary material for: Transcriptome Analysis Reveals Regulation of Gene Expression for Lipid Catabolism in Young Broilers by Butyrate Glycerides
Source: PLoS One. 2016 Aug 10;11(8):e0160751. doi: 10.1371/journal.pone.0160751 (PMC4979964; doi:10.1371/journal.pone.0160751)
Supplement: S8 Table — (DOCX) [file pone.0160751.s008.docx]

**Supplemental Table 8. Treatment specifically expressed genes involved in lipid metabolism in broilers^a^**

|  |  |  |  |  |  |
| --- | --- | --- | --- | --- | --- |
| Treatment | Tissue | Function Annotation | Molecule | No. of genes | *P*-Value |
| BG-Fed group | Jejunum | Activation of cholecalciferol | *CYP24A1* | 1 | 1.18E-02 |
|  |  | Catabolism of  1-alpha, 25-dihydroxy vitamin D3 | *CYP24A1* | 1 | 5.94E-03 |
|  |  | Catabolism of lipid | *CYP24A1, PLA2G2A* | 2 | 2.55E-02 |
|  |  | Hydrolysis of arachidonic acid | *PLA2G2A* | 1 | 1.77E-02 |
|  |  | Hydrolysis of  lysophosphatidylcholine | *PLA2G2A* | 1 | 1.77E-02 |
|  |  | Hydrolysis of  lysophosphatidylethanolamine | *PLA2G2A* | 1 | 5.94E-03 |
|  |  | Hydrolysis of  monohydroperoxy-linoleic acid | *PLA2G2A* | 1 | 5.94E-03 |
|  |  | Hydrolysis of  monohydroxy-arachidonic acid | *PLA2G2A* | 1 | 5.94E-03 |
|  |  | Hydrolysis of phosphatidylglycerol | *PLA2G2A, PLA2G5* | 2 | 2.35E-02 |
|  |  | Liberation of arachidonic acid | *PLA2G2A* | 1 | 1.77E-02 |
|  |  | Oxidation of fat | *UCP3* | 1 | 2.93E-02 |
|  |  | Quantity of sulfatides | *GAL3ST1* | 1 | 1.77E-02 |
|  |  | Release of choline-phospholipid | *PLA2G2A, PLA2G5* | 2 | 2.93E-02 |
|  |  | Release of phosphatidylcholine | *PLA2G2A* | 1 | 2.35E-02 |
|  |  | Secretion of arachidonic acid | *DRD3, PLA2G5* | 2 | 4.65E-02 |
|  |  | Storage of triacylglycerol | *UCP3* | 1 | 2.93E-02 |
|  |  | Synthesis of  1-alpha, 25-dihydroxy vitamin D3 | *CYP24A1* | 1 | 1.18E-02 |
|  |  | Synthesis of galactosylceramide | *GAL3ST1* | 1 | 1.77E-02 |
|  |  | Synthesis of sulfatides | *GAL3ST1* | 1 | 1.18E-02 |
|  |  | Translocation of bile salt | *SLCO1A2* | 1 | 1.77E-02 |
|  |  | Transport of bile salt | *SLCO1A2* | 1 | 2.93E-02 |
|  |  | Transport of taurocholic acid | *SLCO1A2* | 1 | 2.93E-02 |
|  |  | Uptake of bile salt | *SLCO1A2* | 1 | 2.35E-02 |
|  |  | Uptake of paclitaxel | *SLCO1A2* | 1 | 5.94E-03 |
|  | Liver | Abnormal quantity of lipid | *CACNA1B, ELOVL4, GRHL3, NTRK2* | 4 | 2.03E-02 |
|  |  | Concentration of corticosterone | *LEPR, NPY2R, NTRK2, StAR* | 4 | 9.03E-03 |
|  |  | Concentration of lipid | *CACNA1B, EEF1A2, ELOVL4, GHSR, GNAO1,GRHL3, LEPR, NPY2R, NTRK2, PHEX, PTGFR, RAG2, StAR* | 13 | 1.37E-02 |
|  |  | Conversion of pregnenolone | *StAR* | 1 | 4.61E-02 |
|  |  | Metabolism of cholesterol | *CH25H, LEPR, StAR* | 3 | 2.90E-02 |
|  |  | Quantity of  12(S)-hydroxyeicosatetraenoic acid | *LEPR* | 1 | 1.17E-02 |
|  |  | Quantity of  13(S)-hydroxyoctadecadienoic acid | *LEPR* | 1 | 1.17E-02 |
|  |  | Quantity of steroid | *EEF1A2, LEPR, NPY2R, NTRK2,*  *PHEX, PTGFR, RAG2, StAR* | 8 | 2.50E-02 |
|  |  | Synthesis of very long chain fatty acid | *ELOVL4* | 1 | 3.48E-02 |
| BD-Fed group | Jejunum | Concentration of aldosterone | *PVALB, StAR* | 2 | 1.67E-02 |
|  |  | Concentration of cholesterol ester | *CBS, CETP, CYP8B1* | 3 | 4.62E-04 |
|  |  | Concentration of corticosterone | *CETP, NPY2R, StAR* | 3 | 1.47E-02 |
|  |  | Conversion of pregnenolone | *StAR* | 1 | 2.92E-02 |
|  |  | Distribution of cholesterol | *CBS, CETP* | 2 | 1.46E-03 |
|  |  | Distribution of triacylglycerol | *CBS, CETP* | 2 | 5.36E-05 |
|  |  | Elongation of very long chain fatty acid | *ELOVL2* | 1 | 1.47E-02 |
|  |  | Exchange of cholesterol ester | *CETP* | 1 | 7.39E-03 |
|  |  | Exchange of triacylglycerol | *CETP* | 1 | 7.39E-03 |
|  |  | Hydrolysis of cholesterol ester | *CETP* | 1 | 2.92E-02 |
|  |  | Import of cholesterol | *StAR* | 1 | 2.92E-02 |
|  |  | Metabolism of cholesterol | *CETP, CYP8B1, StAR, TNFSF4,* | 4 | 8.43E-03 |
|  |  | Quantity of steroid | *CBS, CETP, CYP8B1, NPY2R, PVALB, StAR* | 6 | 2.32E-02 |
|  |  | Quantity of steroid hormone | *CETP, NPY2R, PVALB, StAR* | 4 | 4.61E-03 |
|  |  | Redistribution of cholesterol ester | *CETP* | 1 | 7.39E-03 |
|  |  | Redistribution of triacylglycerol | *CETP* | 1 | 7.39E-03 |
|  |  | Removal of cholesterol ester | *CETP* | 1 | 7.39E-03 |
|  |  | Synthesis of cholesterol | *CETP, CYP8B1* | 2 | 3.18E-02 |
|  |  | Synthesis of very long chain fatty acid | *ELOVL2* | 1 | 2.20E-02 |
|  |  | Translocation of cholesterol | *STAR* | 1 | 2.92E-02 |
|  |  | Translocation of cholesterol ester | *CETP* | 1 | 7.39E-03 |
|  |  | Translocation of sterol | *CETP, StAR* | 2 | 5.28E-04 |
|  |  | Transmission of cholesterol ester | *CETP* | 1 | 7.39E-03 |
|  |  | Transmission of phosphatidylcholine | *CETP* | 1 | 1.47E-02 |
|  |  | Transmission of sterol | *CETP, StAR* | 2 | 1.10E-03 |
|  |  | Transport of cholesterol | *CETP, StAR* | 2 | 9.35E-03 |
|  |  | Transport of cholesterol ester | *CETP* | 1 | 7.39E-03 |
|  |  | Transport of triacylglycerol | *CETP* | 1 | 2.92E-02 |
|  |  | Uptake of 2-aminoisobutyric acid | *SLC7A10* | 1 | 1.47E-02 |
|  |  | Uptake of lipid | *CETP, CYP8B1, GHRL, SLC7A10, StAR* | 5 | 5.36E-03 |

**^a^** Determined by IPA analysis; n = 2, each sample was a combined sample from three chickens.

Note: *CACNA1B*, Calcium channel, voltage-dependent, N type, alpha 1B subunit (HGNC: 1389); *CBS*, Cystathionine-beta-synthase (HGNC: 1550); *CETP*, Cholesteryl ester transfer protein, plasma (HGNC: 1869); *CH25H*, Cholesterol 25-hydroxylase (HGNC: 1907); *CYP8B1*, Cytochrome P450, family 8, subfamily B, polypeptide 1 (HGNC: 2653); *CYP24A1*, Cytochrome P450, family 24, subfamily A, polypeptide 1 (HGNC: 2602); *DRD3*, Dopamine receptor D3 (HGNC: 3024); *EEF1A2*, Eukaryotic translation elongation factor 1 alpha 2 (HGNC: 3192); *ELOVL2*, ELOVL fatty acid elongase 2 (HGNC: 14416); *ELOVL4*, ELOVL fatty acid elongase 4 (HGNC: 14415); *GAL3ST1*, Galactose-3-O-sulfotransferase 1 (HGNC: 24240); *GHSR*, Growth hormone secretagogue receptor (HGNC: 4267); *GHRL*, Gghrelin/obestatin prepropeptide (HGNC: 18129); *GNAO1*, Guanine nucleotide binding protein (G protein), alpha activating activity polypeptide O (HGNC: 4389); *GRHL3*, Grainyhead-like 3 (Drosophila) (HGNC: 25839); *LEPR*, Leptin receptor (HGNC: 6554); *NPY2R*, Neuropeptide Y receptor Y2 (HGNC: 7957); *NTRK2*, Neurotrophic tyrosine kinase, receptor, type 2 (HGNC: 8032); *PHEX*, Phosphate regulating endopeptidase homolog, X-linked (HGNC: 8918); *PLA2G2A*, Phospholipase A2, group IIA (platelets, synovial fluid) (HGNC: 9031); *PLA2G5*, Phospholipase A2, group V (HGNC: 9038); *PTGFR*, Prostaglandin F receptor (FP) (HGNC: 9600); *PVALB*, Parvalbumin (HGNC: 9704); *RAG2*, Recombination activating gene 2 (HGNC: 9832); *SLC7A10*, Solute carrier family 7 (neutral amino acid transporter light Chain, asc system), member 10 (HGNC: 11058); *SLCO1A2*, Solute carrier organic anion transporter family, member 1A2 (HGNC: 10956); *StAR*, Steroidogenic acute regulatory protein (HGNC: 11359); *UCP3*, Uncoupling protein 3 (mitochondrial, proton carrier) (HGNC: 12519); *TNFSF4*, Tumor necrosis factor (ligand) superfamily, member 4 (HGNC: 11934).
